# Associations of childhood body composition trajectories and adolescent body dissatisfaction: Longitudinal evidence from the UK Millennium Cohort Study

**DOI:** 10.1371/journal.pone.0339555

**Published:** 2026-10-14

**Authors:** Madelaine Davies Kellock, Francesca Solmi, Anne McMunn, Yvonne Kelly

**Affiliations:** 1 Department of Epidemiology & Public Health, Institute of Epidemiology & Health Care, UCL, London, United Kingdom; 2 Department of Epidemiology & Applied Clinical Research, Division of Psychiatry, UCL, London, United Kingdom; Universidade do Estado do Rio de Janeiro, BRAZIL

## Abstract

Body dissatisfaction is common, particularly among those with higher BMIs. Issues with BMI as an adiposity measure may limit our understanding of how body weight is associated with body dissatisfaction, particularly given gendered body ideals. We investigated the association of childhood trajectories of BMI, fat mass index (FMI), and fat-free mass index (FFMI) with body dissatisfaction at age 14, and sex differences in these associations. This was a secondary analysis of UK Millennium Cohort Study data. Our exposures were trajectories of BMI (3−14 years), FMI and FFMI (7−14 years), modelled using latent class growth analysis. Our outcome was body dissatisfaction at 14 years. We used univariable and multivariable linear regression models to investigate associations and interaction terms to test for sex differences. Among 13,025 participants (49.2% female; 84.4% White), we found three BMI, FMI, and FFMI trajectories (low, moderate, high). Participants with moderate (adjusted mean difference [aMD] 0.32, 95% confidence interval [CI] 0.24, 0.39) and high (aMD 0.71, 95% CI 0.55, 0.86) BMI trajectories and those with moderate (aMD 0.33, 95% CI 0.25, 0.41) and high (aMD 0.73, 95% CI 0.57, 0.89) FMI trajectories had higher body dissatisfaction scores than those with low trajectories. We did not find evidence that these associations differed by sex. For FFMI trajectories, boys with high (aMD 0.31, 95% CI 0.17, 0.46), but not moderate (aMD 0.07, 95% CI −0.03, 0.17) trajectories had greater body dissatisfaction. In girls, mean differences by FFMI trajectory were consistent with adiposity results. BMI and FMI trajectories are similarly associated with body dissatisfaction when FMI is estimated using bioelectrical impedance analysis. Both boys and girls may be susceptible to weight-related body dissatisfaction in adolescence. Further research is needed to understand how muscularity is associated with body dissatisfaction.

## Introduction

Body dissatisfaction has been rising in young people over the past few decades [1], which is concerning given its association with subsequent mental health problems, including depression and eating disorders [2,3]. Through a range of sources from an early age [4,5], young people are exposed to messaging which values some body types and stigmatises others [6]. In many Western contexts, thinness in women and lean muscularity in men are seen as socially desirable, while overweight is stigmatised [7]. These ideals can become internalised and lead to body dissatisfaction.

Previous cross-sectional research has shown that higher body weight is associated with body dissatisfaction and, consistent with gendered body ideals, there appear to be sex differences in this association [8]. In girls, increasing weight is linearly associated with greater body dissatisfaction, while in boys, the association is J-shaped, as both those with low and high weight have higher body dissatisfaction [8]. However, prior research has almost exclusively used BMI to indicate weight status as a proxy for adiposity. BMI measures total body weight and cannot distinguish between its component parts, namely fat and fat-free mass. As such, individuals with the same BMI value can have different proportions of fat mass, potentially leading to misclassification of weight status. Some evidence suggests that BMI may be a poor proxy for adiposity, particularly in younger children [9] and in those with non-overweight BMIs, which is the majority of children [10]. Fat and fat-free mass may be associated with body dissatisfaction, and their associations with the latter may differ in boys and girls given the social desirability of muscularity in boys.

Few cross-sectional studies have investigated the associations of fat and fat-free mass with body dissatisfaction. These have been predominantly conducted in athletes using body fat percentage to measure adiposity [11]. Body fat percentage, however, may be elevated due to high adiposity or low lean mass [12] and so, like BMI, cannot convey the nuanced associations that fat and fat-free mass may have with body dissatisfaction. A small number of cross-sectional studies have utilised fat and fat-free mass measures, with some finding no association with body dissatisfaction for either fat mass or fat-free mass [13,14], and some finding that both higher fat mass [15] and higher fat-free mass are associated with increased body dissatisfaction [15,16]. These studies were conducted in athletes and sport science students, who may differ from the general population both in terms of body composition and body dissatisfaction, limiting generalisability. Furthermore, due to their cross-sectional designs, causal inference is limited as individuals experiencing body dissatisfaction may attempt to alter their weight [3].

To our knowledge, only one longitudinal, general population study has investigated the association between fat and fat-free mass and body dissatisfaction [17], using polygenic scores as instrumental variables for body composition, and finding that higher polygenic scores for both fat and fat-free mass are associated with greater body dissatisfaction in adolescence. As this finding contrasts with the theory that higher muscle mass is socially desirable in boys, this association warrants further investigation.

Previous studies have established that there are distinct trajectories of BMI development across childhood [18–20] and that children with increasing BMI trajectories report higher body dissatisfaction at age 11 [18]. There has been little investigation into trajectories of fat and fat-free mass across childhood [21]. Using trajectories of body composition, we can disentangle the potentially distinct associations of fat and fat-free mass across childhood and body dissatisfaction in boys and girls, acknowledging the lifecourse nature of weight development. This knowledge may help in designing interventions to promote good mental health in adolescence, given the links between body dissatisfaction and subsequent mental health problems [2,3]. Further, this may suggest limitations when using BMI as a proxy for adiposity, which is pertinent due to BMI’s ubiquity across research.

Therefore, we aimed to investigate whether trajectories of BMI, fat mass index (FMI) and fat-free mass index (FFMI) are differentially associated with body dissatisfaction at age 14, and whether these associations differ by sex. Based on existing knowledge and theory around gendered body ideals, we hypothesised that higher trajectories of adiposity (measured by FMI) across childhood would be associated with greater body dissatisfaction in adolescence, and that the magnitude of association would be greater in girls relative to boys. We also hypothesised that higher trajectories of FFMI, as a proxy for muscularity, would be associated with lower body dissatisfaction in boys only.

## Methods

### Sample

This study was a secondary analysis of data from the Millennium Cohort Study, a prospective longitudinal study of children living in the UK who were born between September 2000 and January 2002. The Millennium Cohort Study is a socially and ethnically diverse cohort, with baseline data collection at age 9 months, with subsequent sweeps at ages 3, 5, 7, 11, 14, and 17 years. The Millennium Cohort Study obtained ethical approval from the National Health Service Research Ethics Committee prior to all sweeps; at age 14, this was the London–Central MREC [13/LO/1786] (see [22] for further details). Written informed consent was sought from parents and, from age 11, the child cohort members, for their participation in the study. Further details of the cohort, including sampling methods can be found elsewhere [23]. We accessed deidentified data on 8^th^ March 2021. For this study, we included participants who had at least one measurement available for each aspect of body composition data up to age 14. In twin pairs, we excluded one twin at random to avoid potential biases arising from shared genetic and environmental factors. As we wanted to compare across multiple measures of body composition, and some were not measured at earlier sweeps, we restricted our analyses to participants with data on all body composition measures (see Exposures).

### Outcome

Body dissatisfaction was measured at age 14 years. In a self-reported questionnaire, participants were asked: “On a scale of 1 to 7, where 1 means completely happy and 7 means not at all happy, how do you feel about the way you look?”. This was recoded to range from 0 to 6, where higher scores indicate greater body dissatisfaction. This item has been widely used as an indicator of body dissatisfaction in prior literature [18,24].

### Exposures

BMI was calculated from measured height and weight at ages 3, 5, 7, 11, and 14 years, which were measured without shoes or outdoor clothing by a trained interviewer [25]. Height was measured to the nearest 0.1 cm using a Leicester stadiometer and weight to the nearest 0.1 kg using Tanita scales. Additionally, from age 7, Tanita scales (BF-522W) measured body fat percentage using bioelectrical impedance analysis to the nearest 0.1%. In bioelectrical impedance analysis, a weak electrical current is sent around the body from foot to foot to estimate the proportion of fat. Age, height and sex were recorded on the scales to aid estimation, calculated using proprietary equations determined by the manufacturer and based on large, multi-ethnic population studies.

Using measured height and weight, we calculated BMI as $weight÷({height}^{\text{2}})$ and using body fat percentage we calculated fat mass as (body fat percentage÷100)×weight and fat-free mass as weight−fat mass, allowing us to estimate FMI as $fat\;mass÷\left({height}^{\text{2}}\right)$ and FFMI as $fatfree\;mass÷{(height}^{\text{2}})$. Range checks incorporated in interviewer scripts highlighted implausible values to improve accuracy of data collection. Additionally, we checked for extreme observations and excluded values which were more than 5 Standard Deviations above or below the study mean. Methods used to model trajectories are described in Data analyses.

### Confounders

Potential confounding variables were selected based on existing evidence of their association with weight development and mental health outcomes and were measured when the participants were 9 months of age, unless otherwise specified. These were: child sex at birth and ethnicity, indicators of family socioeconomic position (family income, parent education and occupational class), perinatal factors (child birthweight, maternal pre-pregnancy BMI, breastfeeding status, and maternal use of cigarettes or alcohol during pregnancy), and maternal psychological distress when the child was aged 3 years. We also adjusted for child socioemotional difficulties at age 3, measured by the parent-reported Strengths and Difficulties Questionnaire (SDQ) total score [26]. Further detail on confounders, including a directed acyclic graph outlining our causal assumptions, can be found in the S1 Appendix, page 1).

### Data analyses

All analyses were conducted in Stata 17 (Stata Statistical Software: Release 17. College Station, TX: StataCorp LLC).

#### Trajectory analysis.

We modelled trajectories of BMI, FMI and FFMI for children with at least one valid measurement for each of the body composition measures, employing latent class growth analysis [27,28] using maximum likelihood estimation to account for missing data. Starting with a two-class model and increasing the number of classes, we modelled different combinations of polynomial shapes. The optimal polynomial shapes and number of classes was chosen based on goodness of fit (Akaike Information Criterion) and neatness of classification (Entropy Index). These were considered in addition to the ability of each model to identify distinct and meaningful patterns in the data, while maintaining group sample sizes sufficiently large to ensure adequate statistical power for subsequent analyses. After selecting the optimal models, we assigned participants to their most likely trajectory. Full details of latent class trajectory analysis can be found in the S1 Appendix (page 3).

#### Descriptive analyses.

Among participants with valid exposure data, we described the overlap between trajectories of different body composition measures using proportions. We described characteristics of the overall sample, as well as the distribution of trajectory membership across confounding variables using frequencies and proportions.

#### Missing data.

Among participants with complete exposure data, we explored frequencies and proportions of missing data on potential confounders, as well as differences between participants with valid and missing outcome data. Missing data on outcomes and confounders were then imputed using multiple imputation by chained equations, under the assumption that data are missing at random. Fifty datasets were imputed using all analysis variables and additional auxiliary variables to improve precision (see S1 Appendix, page 4).

#### Regression analyses.

To investigate the association between childhood body composition trajectory and body dissatisfaction at age 14, we used univariable and multivariable linear regression models with separate adjustment for child sex and ethnicity (Model 1), indicators of family socioeconomic position (Model 2), perinatal factors (Model 3), maternal psychological distress (Model 4) and child socioemotional difficulties at age 3 (Model 5). We then ran a fully adjusted model with all confounders in models 1–5, before including an exposure*sex interaction to investigate sex differences in the association. If there was evidence of interaction, we reported sex-stratified results.

All regression models were run on a sample with complete exposures with imputed outcomes and confounders. We also included sampling weights to account for the stratified sampling methods.

#### Sensitivity analyses.

We ran two sensitivity analyses. First, we repeated unadjusted regression models in the full sample with BMI trajectory data to investigate whether our estimates were impacted by restricting to those with data on both BMI and other body composition measures. Second, we repeated our univariable and multivariable regression analyses in a sample with complete data on exposures, outcomes and confounders.

## Results

### Sample

Of 18,540 participants, 13,025 (70.3%) had valid trajectory group data for all exposures. Approximately half of the participants were male (50.8%), the majority were of White ethnicity (84.4%) and most had a parent educated to degree level or equivalent (42.2%, see **Table 1**).

**Table 1:** Characteristics of sample with valid exposure data and the distribution of trajectory membership among characteristics (N = 13025^a^). Proportions of sample with observed data (unweighted).

Characteristic	N (%)	BMI Trajectory N (%)			FMI Trajectory N (%)			FFMI Trajectory N (%)		
		Low	Moderate	High	Low	Moderate	High	Low	Moderate	High
Total (%)		64.3	29.0	6.7	68.6	25.2	6.2	44.9	45.9	9.2
**Sex at birth**										
Male	6612 (50.8)	67.6	26.5	5.9	79.6	16.2	4.2	34.1	51.8	14.1
Female	6413 (49.2)	60.9	31.6	7.4	57.2	34.6	8.2	56.1	39.8	4.1
**Ethnicity**										
White	10968 (84.4)	64.6	29.2	6.2	69.8	24.6	5.6	43.4	47.2	9.4
Mixed	346 (2.7)	65.6	25.4	9.0	67.3	23.7	9.0	46.0	44.2	9.8
Indian	312 (2.4)	68.6	26.0	5.4	64.1	27.9	8.0	62.8	34.6	2.6
Pakistani or Bangladeshi	828 (6.4)	62.2	27.9	9.9	59.5	30.6	9.9	58.3	35.0	6.7
Black or Black British	383 (2.9)	52.0	36.0	12.0	57.7	30.6	11.7	37.9	47.2	14.9
Other, including Chinese and Arab	160 (1.2)	70.0	23.1	6.9	67.5	26.9	5.6	57.5	36.9	5.6
**Family income at 9 months**										
Most disadvantaged	2804 (21.6)	60.4	30.3	9.3	63.8	27.1	9.1	43.9	45.5	10.6
Second	2786 (21.4)	62.4	30.0	7.6	65.5	27.4	7.1	44.5	45.2	10.3
Third	2526 (19.4)	62.4	31.1	6.5	67.3	26.5	6.2	43.0	47.5	9.5
Fourth	2521 (19.4)	64.7	29.7	5.6	70.0	25.3	4.7	43.6	47.7	8.7
Least disadvantaged	2355 (18.1)	72.8	23.5	3.7	77.5	19.2	3.3	49.9	43.6	6.5
Highest parental education level at 9 months										
None or overseas	1403 (10.8)	60.1	29.9	10.0	62.3	28.2	9.5	47.2	41.8	11.0
NVQ Level 1	775 (5.9)	62.6	29.9	7.5	67.6	25.7	6.7	46.2	43.6	10.2
NVQ Level 2	3199 (24.6)	60.6	31.4	8.0	64.5	27.7	7.8	42.0	47.8	10.2
NVQ Level 3	2151 (16.5)	63.6	29.2	7.2	68.3	25.5	6.2	44.2	45.3	10.5
NVQ Level 4	4593 (35.3)	67.2	27.9	4.9	71.8	23.5	4.7	45.2	47.2	7.6
NVQ Level 5	896 (6.9)	72.5	23.9	3.6	77.5	19.7	2.8	50.9	42.2	6.9
**Highest parental occupational class at 9 months**										
Not in work	2924 (22.5)	60.5	30.6	8.9	64.2	26.9	8.9	44.0	45.0	10.9
Routine or semi-routine	1900 (14.6)	60.7	30.5	8.8	63.6	28.3	8.1	42.8	46.6	10.6
Lower supervisory or technical	1043 (8.0)	59.2	33.2	7.6	64.6	27.9	7.5	41.6	48.4	10.0
Small employers or self-employed	863 (6.7)	63.6	29.8	6.6	67.3	26.4	6.3	44.8	45.4	9.7
Intermediate occupations	1199 (9.2)	66.3	27.5	6.2	70.1	24.8	5.2	45.8	45.9	8.3
Managerial or professional	5050 (38.9)	68.5	27.1	4.4	73.6	22.6	3.8	46.8	45.6	7.6
**Birthweight**										
Low birthweight (≤2.5 kg)	890 (6.9)	68.7	24.9	6.4	67.9	24.8	7.3	55.8	36.5	7.6
Birthweight >2.5 kg	12106 (93.1)	64.0	29.3	6.7	68.6	25.3	6.1	44.1	46.6	9.3
**Maternal pre-pregnancy BMI**										
BMI < 25	8416 (70.3)	71.4	24.6	4.0	74.8	21.5	3.7	50.4	43.2	6.4
BMI 25–29.9	2465 (20.6)	53.6	36.7	9.7	60.0	31.2	8.8	35.6	51.6	12.8
BMI ≥ 30	1083 (9.1)	38.2	43.1	18.7	45.9	37.2	16.9	24.3	53.6	22.1
**Breastfed**										
No	3870 (29.8)	60.8	31.0	8.2	64.7	27.4	7.9	42.5	47.2	10.3
Yes	9135 (70.2)	65.8	28.2	6.0	70.2	24.3	5.5	46.0	45.3	8.7
**Cigarette use in pregnancy**										
None	9455 (72.6)	65.8	28.3	5.9	69.4	24.9	5.7	46.5	45.2	8.4
Any	3564 (27.4)	60.5	31.0	8.5	63.8	27.2	9.0	36.4	49.8	13.8
**Alcohol use in pregnancy**										
None	9020 (69.4)	63.0	29.7	7.3	67.0	26.0	7.0	45.1	45.3	9.6
Any	3982 (30.6)	67.4	27.3	5.3	72.1	23.5	4.3	44.6	47.1	8.3
**Maternal psychological distress at 3 years**										
Low (<5)	8591 (75.0)	67.0	27.1	5.9	69.6	24.8	5.6	44.3	46.8	8.9
Moderate (5–11)	2472 (21.6)	65.2	27.2	7.6	66.5	26.3	7.2	42.4	46.8	10.8
Severe (≥12)	388 (3.4)	65.5	26.0	8.6	65.7	26.0	8.3	43.0	47.4	9.5
**Child total SDQ score at 3 years**										
Close to average (≤13)	9591 (79.0)	64.8	29.1	6.1	69.2	25.0	5.8	45.3	46.0	8.7
Slightly raised (14–16)	1276 (10.5)	61.4	30.5	8.1	67.0	26.3	6.7	40.8	48.4	10.7
High (17–19)	678 (5.6)	63.1	28.9	8.0	68.6	23.7	7.7	42.6	45.1	12.3
Very high (≥20)	593 (4.9)	61.9	28.7	9.4	67.6	24.0	8.4	44.2	44.3	11.5

a Ns may not total 13025 due to missing data; see Supplementary Information for more on missing data.

### Body composition trajectories

For each measure of body composition, the three-trajectory model, including a low, a moderate, and a high trajectory group (Fig 1) was considered optimal. Full results of trajectory modelling can be found in the S1 Appendix (page 5). The majority of participants were assigned to a low trajectory of BMI (64.3%) and FMI (68.6%), with 29.0% (BMI) and 25.2% (FMI) of participants assigned to a moderate trajectory and 6.7% (BMI) and 6.2% (FMI) to a high trajectory (**Table 1**). Roughly equal proportions of participants had low and moderate trajectories of FFMI (44.9% and 45.9%, respectively), while 9.2% had high trajectories. There was large overlap between BMI and adiposity trajectory membership, but less so for FFMI (**Table 2**).

**Table 2:** Overlap between body composition trajectory membership.

		BMI Trajectory (%)			FMI Trajectory (%)			FFMI Trajectory (%)		
BMI Trajectory	%	Low	Moderate	High	Low	Moderate	High	Low	Moderate	High
Low	64.3				95.2	4.8	0.0	66.7	33.1	0.2
Moderate	29.0				25.1	71.6	3.3	6.8	76.7	16.6
High	6.7				0.8	20.9	78.3	1.3	35.1	63.7
FMI Trajectory										
Low	68.6	89.3	10.6	0.1				59.3	37.1	3.6
Moderate	25.2	12.2	82.3	5.5				16.4	69.5	14.1
High	6.2	0.1	15.7	84.2				2.2	46.7	51.1
FFMI Trajectory										
Low	44.9	95.4	4.4	0.2	90.5	9.2	0.3			
Moderate	45.9	46.4	48.5	5.1	55.4	38.3	6.3			
High	9.2	1.6	52.3	46.1	26.9	38.7	34.4			

**Fig 1:**
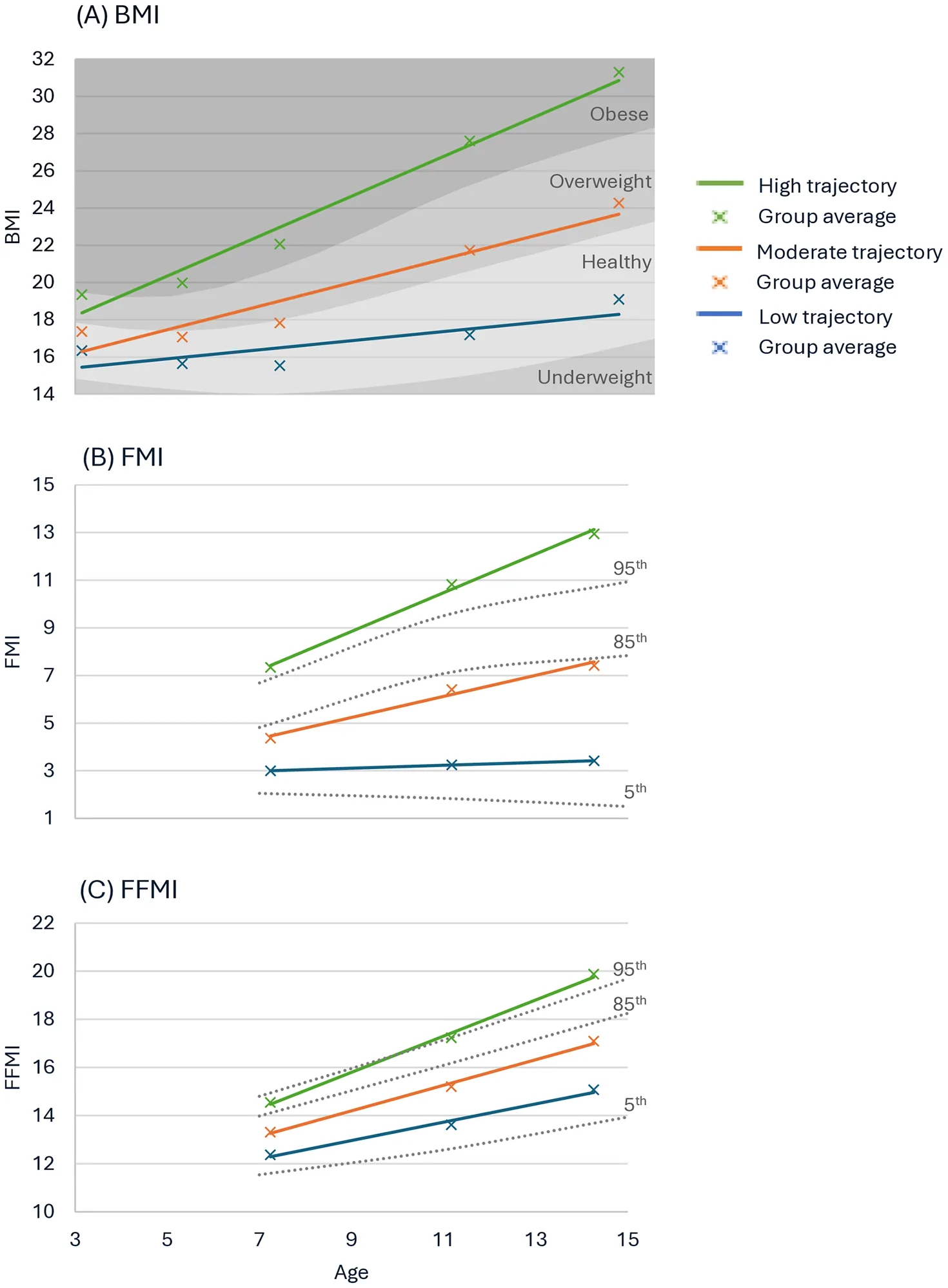
Body composition trajectories for (A) BMI, (B) FMI, (C) FFMI. Crosses show average data points for each group with lines representing the estimated trajectories. For BMI, shaded areas show the International Obesity Task Force thresholds for categorising child BMI. For FMI and FFMI dashed lines reflect centiles within the MCS sample.

A greater proportion of girls, participants of mixed, Pakistani, Bangladeshi, or Black ethnicity, and from more socioeconomically disadvantaged backgrounds had higher trajectories of BMI and FMI across childhood (**Table 1**). Higher maternal pre-pregnancy BMI, psychological distress, and participant SDQ score at age 3 were all associated with higher participant BMI and FMI trajectories. In contrast to these exposures, a greater proportion of boys and participants from more socioeconomically advantaged backgrounds had higher trajectories of FFMI (**Table 1**).

### Missing data

Among those with valid exposure data, 5,092 were missing data on outcomes or confounders. A greater proportion of those with missing data were male, from ethnic minority backgrounds, more socioeconomically disadvantaged, and had greater socioemotional difficulties at age 3 as well as mothers with higher levels of psychological distress (see S1 Appendix, page 13).

### Association between childhood body composition trajectories and body satisfaction at age 14

In unadjusted analyses (**Table 3**), there was evidence that children with moderate (mean difference [MD] 0.41, 95% Confidence Intervals [CI] 0.34, 0.49) and high (MD 0.87, 95% CI 0.71, 1.02) trajectories of BMI had higher body dissatisfaction scores in adolescence, compared to those with low trajectories. Patterns of association were similar for FMI, though mean differences were larger for both moderate (MD 0.59, 95% CI 0.51, 0.67) and high (MD 1.02, 95% CI 0.85, 1.18) trajectories. In fully adjusted models, there remained strong evidence of associations for both BMI (moderate: adjusted MD 0.32, 95% CI 0.24, 0.39; high: aMD 0.71, 95% CI 0.55, 0.86) and FMI (moderate: aMD 0.33, 95% CI 0.25, 0.41; high: aMD 0.73, 95% CI 0.57, 0.89), and mean differences were consistent across the two measures. There was no evidence of sex differences in the association of BMI (interaction test p value for moderate trajectory = 0.216; high p = 0.140) or FMI (moderate p = 0.968; high p = 0.207) and body dissatisfaction.

**Table 3:** Results of univariable and multivariable linear regression analysis of the association between body composition trajectory and body dissatisfaction at age 14 in participants with valid exposure data and imputed outcomes and confounders (sampling weights applied).

Model	*Mean difference in body dissatisfaction at age 14 years (95% confidence interval)*								
	BMI Trajectory			FMI Trajectory			FFMI Trajectory		
	Low	Moderate	High	Low	Moderate	High	Low	Moderate	High
Model 0(unadjusted)	ref	0.41 (0.34, 0.49)	0.87 (0.71, 1.02)	ref	0.59 (0.51, 0.67)	1.02 (0.85, 1.18)	ref	0.07 (−0.00, 0.15)	0.17 (0.03, 0.30)
Model 1 (0 + child characteristics^a^)	ref	0.36 (0.28, 0.43)	0.80 (0.66, 0.96)	ref	0.38 (0.30, 0.46)	0.83 (0.68, 1.00)	ref	0.27 (0.19, 0.34)	0.59 (0.45, 0.72)
Model 2 (0 + family SEP^b^)	ref	0.40 (0.33, 0.48)	0.84 (0.68, 1.00)	ref	0.58 (0.50, 0.66)	0.99 (0.83, 1.15)	ref	0.06 (- 0.01, 0.14)	0.14 (0.01, 0.27)
Model 3 (0 + perinatal factors^c^)	ref	0.40 (0.31, 0.47)	0.80 (0.64, 0.96)	ref	0.56 (0.48, 0.65)	0.96 (0.80, 1.12)	ref	0.04 (−0.04, 0.11)	0.07 (−0.07, 0.20)
Model 4 (0 + maternal psychological distress^d^)	ref	0.41 (0.34, 0.49)	0.85 (0.69, 1.01)	ref	0.58 (0.51, 0.66)	1.00 (0.84, 1.17)	ref	0.07 (−0.00, 0.15)	0.16 (0.03, 0.29)
Model 5 (0 + child socioemotional difficulties^e^)	ref	0.41 (0.34, 0.49)	0.86 (0.70, 1.01)	ref	0.59 (0.51, 0.67)	1.01 (0.85, 1.17)	ref	0.07 (−0.01, 0.14)	0.15 (0.02, 0.28)
Model 6 (0 + all confounders in Models 1–5)	ref	0.32 (0.24, 0.39)	0.71 (0.55, 0.86)	ref	0.33 (0.25, 0.41)	0.73 (0.57, 0.89)	ref	0.23 (0.15, 0.30)	0.48 (0.34, 0.62)
Exposure × sex interactionp value	ref	0.216	0.140	ref	0.968	0.207	ref	<0.001	0.003
Mean difference in boys (N = 6612)	ref	–	–	ref	–	–	ref	0.07 (−0.03, 0.17)	0.31 (0.17, 0.46)
Mean difference in girls (N = 6413)	ref	–	–	ref	–	–	ref	0.36 (0.26, 0.47)	0.79 (0.47, 1.10)

^a^Child sex at birth, ethnicity.

^b^Family income quintile, parental educational qualification level, parental occupational class.

^c^Child birthweight, maternal pre-pregnancy BMI, breastfeeding, cigarette and alcohol use in pregnancy.

^d^Maternal psychological distress score at 3 years old.

^e^Child total SDQ score at 3 years old.

In contrast, for FFMI trajectories, there was evidence in unadjusted models of smaller mean differences in body dissatisfaction among those with moderate (MD 0.07, 95% CI −0.00, 0.15) and high FFMI (MD 0.17, 95% CI 0.03, 0.30) compared to the low trajectory group (**Table 3**). When adjusting for child sex and ethnicity, the magnitude of these mean differences increased (moderate: aMD 0.27, 95% CI 0.19, 0.34; high: aMD 0.59, 95% CI 0.45, 0.72), such that these were more consistent with associations seen for BMI and FMI trajectories. In the fully adjusted model, there was strong evidence that participants with moderate (aMD 0.23, 95% CI 0.15, 0.30) and high (aMD 0.48, 95% CI 0.34, 0.62) FFMI trajectories had higher body dissatisfaction than those with low FFMI trajectories, but mean differences were smaller than those seen for adiposity measures. There was strong evidence that these associations differed by sex (interaction test p value for moderate trajectory <0.001; high p = 0.003).

In boys, only those with high FFMI trajectories had greater body dissatisfaction (moderate: aMD 0.07, 95% CI −0.03, 0.17; high: aMD 0.31, 95% CI 0.17, 0.46). In girls, the association was consistent with those seen for BMI and FMI (moderate FFMI: aMD 0.36, 95% CI 0.26, 0.47; high: aMD 0.79, 95% CI 0.47, 1.10).

### Sensitivity analyses

When we repeated regression models in a sample of participants with valid BMI trajectory data (N = 14,765), results were consistent with those of the main analysis. Similarly, when we repeated our analyses in a sample with complete data on exposures, outcomes and confounders, we found that associations and results of interaction tests were similar to those in the main analysis. See S1 Appendix (page 17) for full results.

## Discussion

In this study we found distinct trajectories of BMI, FMI and FFMI. Our results suggest that higher childhood trajectories of adiposity, measured by BMI or FMI, were associated with high levels of body dissatisfaction in adolescence. There was no evidence that this association differed by sex, which was in contrast to our initial hypothesis. However, we did find evidence that the association between FFMI trajectory and body dissatisfaction differed by sex. We had hypothesised that a negative association would be observed in boys due to the social desirability of muscularity; instead, we found that boys with high FFMI trajectories had greater body dissatisfaction than those with low FFMI, but mean differences were smaller than adiposity estimates. In girls, the association between FFMI and body dissatisfaction was consistent with adiposity measures.

### Limitations

Body dissatisfaction was measured using a single item measure of global appearance satisfaction. Although this has not been validated against body dissatisfaction scales, it has been used widely in prior research as a proxy for body dissatisfaction [1,18,24,29]. Nevertheless, global appearance dissatisfaction is conceptually distinct from the multidimensional construct of body dissatisfaction and cannot distinguish dissatisfaction with specific aspects of body shape or size, which may be particularly pertinent in understanding sex differences. For example, multidimensional body dissatisfaction scales, such as those used in other cohorts [30], can capture specific dissatisfaction with body fat or muscularity. Adolescents may feel satisfied with some aspects of their appearance and not with others, though this nuance is not captured with our global appearance dissatisfaction score, limiting our interpretation.

Body composition data was measured using bioelectrical impedance analysis, rather than the gold standard dual x-ray absorptiometry scans. Our body composition estimates from bioelectrical impedance analysis are based on manufacturer-derived equations and device-specific validations appear to have largely been based on data from White children (e.g., [31]). As dual x-ray absorptiometry was not used, this limited our investigation of distinct components of body composition to fat and fat-free mass. We were unable to further break down fat-free mass, for instance into lean soft tissue and bone components which would be possible with dual x-ray absorptiometry. In this study, we were interested in fat-free mass as it may partly reflect muscularity. However, factors other than lean mass may contribute to raised fat-free mass, such as elevated bone mass [32], or as an adaptation to elevated fat mass [33]. As a result, higher FFMI trajectory groups are likely to capture multiple phenotypes beside higher muscularity and should therefore not be interpreted as reflecting the association of muscularity and body dissatisfaction.

We assigned participants to their most likely trajectory group using modal class assignment. Although our entropy indices indicated adequate classification, there is likely to be some uncertainty and potential misclassification, particularly with FFMI trajectories where entropy values were lower, which may bias associations with body dissatisfaction.

We created indices of body composition measures by dividing each indicator by height squared, as is standard practice; but recent evidence suggests that this may not sufficiently account for the correlation between weight and height, particularly when using fat and fat-free mass [34]. Therefore, there may be residual confounding by height, and this could differentially impact girls and boys as taller stature is more socially desirable in men. Further, although we adjusted for a wide range of child and family characteristics, there may be residual confounding by imperfectly measured, unmeasured or unknown factors. As we modelled trajectories of body composition from early childhood, we could not control for pubertal status, as we considered it likely to be on the causal pathway given evidence that higher childhood adiposity is associated with earlier onset of puberty [35]. Nevertheless, as the pubertal transition results in changes to body composition, this may have influenced measures of body composition in late childhood and early adolescence which we incorporated into our trajectory modelling.

Finally, although we imputed missing data, attrition was higher among disadvantaged groups, a greater proportion of whom had higher adiposity trajectories. We included auxiliary variables in our imputation model and found similar results using samples with imputed and complete data, however this patterning of missing data may have impacted our imputation and estimates of the association with body dissatisfaction.

### Interpretation of findings

Based on theory of the gendered nature of sociocultural body ideals, we had expected to find that higher adiposity in girls and lower FFMI in boys are associated with greater body dissatisfaction. Instead, we found that high FFMI was associated with greater body dissatisfaction in both sexes and that the magnitude of this association was greater in girls than in boys, consistent with a previous study [17]. High FFMI could capture a range of different physiological profiles, such as high muscularity, adaptive increases in lean mass as a result of higher adiposity [33], or rapid growth during puberty [36]. It is plausible that high FFMI in these different scenarios may be associated with dissatisfaction with different aspects of body size or shape which are all captured by a global body dissatisfaction score in our study. Within this, there may also be sex differences in the aspects of body size and shape which contribute to overall body dissatisfaction, which we were not able to test as a consequence of using a global dissatisfaction score. Thus, our findings may suggest that high fat-free mass could have different social meanings and subjective body experiences which may vary across developmental stages and sexes. This warrants further investigation using methods of measuring body composition which can distinguish lean mass, which may be socially desirable in men and boys, from other contributors to high fat-free mass. Coupled with body dissatisfaction scales which evaluate different aspects of appearance, this may lead to greater understanding of different risk factors for body dissatisfaction in girls and boys.

We found considerable overlap between BMI and FMI trajectories, as well as in their associations with body dissatisfaction in adolescence. This may suggest that, within this cohort and at these ages, BMI trajectory largely approximates adiposity trajectory at the population level, as estimated by bioelectrical impedance analysis-derived FMI. Effect sizes were fairly modest, representing approximately 12% higher body dissatisfaction scores in those with high BMI or FMI trajectories compared to those with low trajectories, given the 6-point scale used. However, these may translate to large shifts in the distribution of body dissatisfaction at the population level [37]. Given strong evidence of its association with later mental health problems [2,3], this is concerning.

We did not find evidence that the association between adiposity trajectory and body dissatisfaction differed by sex. This is somewhat consistent with previous cross-sectional studies using BMI, which have shown that sex differences are present at lower BMI values, but both girls and boys with higher BMIs are more dissatisfied [8]. Other studies have found a greater magnitude of association among girls, but a positive association in both sexes (e.g., [24]). It is possible that earlier in adolescence there are differences by sex, but by mid-adolescence, as in our study, these differences may disappear, perhaps as more boys have entered puberty. The absence of sex differences in our study may suggest that both boys and girls are susceptible to body dissatisfaction, which may arise because of societal weight stigma although we did not test this mechanism in our study.

Young people are exposed to weight stigma from many sources, including in interpersonal relationships and via various forms of media [6,38]. Additionally, public health strategies aimed at reducing childhood overweight often target primary school-aged children. The statutory primary school curriculum in England includes learning about the risks of obesity and an understanding of calories and other nutritional content [39]. The National Child Measurement Programme has also seen the introduction of children being weighed in school in the first and final years of primary school [40], and a report from a recent Select Committee inquiry into body image called for annual health assessments in schools including weight measurements [41], implying that by identifying and reducing childhood overweight, body image concerns may be prevented. Although we did not measure this in our study, it is possible that these strategies’ focus on weight could contribute to increased stigma and lead to feelings of guilt and shame in children with higher weights.

## Conclusion

Body dissatisfaction is often considered to only affect women and girls, yet it has been rising in adolescent boys [1,42]. Our findings suggest that both girls and boys with higher childhood adiposity are at risk of body dissatisfaction in adolescence. Given links between body dissatisfaction and subsequent mental health problems [2,3], our findings invite action to reduce young people’s exposure to weight-stigmatised messaging. In this cohort, BMI trajectory approximated FMI trajectory and its association with body dissatisfaction, but FFMI trajectories were more distinct and their associations with body dissatisfaction differed among boys. Further research should investigate the associations between components of fat-free mass, including lean mass, and body dissatisfaction using scales that capture satisfaction with different aspects of body size and shape.

## Supporting information

S1 Appendix Appendix supplement. (DOCX)
