# Supplementary material for: Associations of childhood body composition trajectories and adolescent body dissatisfaction: Longitudinal evidence from the UK Millennium Cohort Study
Source: PLoS One. 2026 Oct 14;21(10):e0339555. doi: 10.1371/journal.pone.0339555 (PMC13667782; doi:10.1371/journal.pone.0339555)
Supplement: Supplementary file 1 [file pone.0339555.s002.pdf]

Review Comments to the Author

This manuscript addresses an important and timely question by examining longitudinal associations between childhood body composition trajectories and body dissatisfaction in adolescence, using data from the UK Millennium Cohort Study. The study is thoughtfully designed and makes a valuable contribution by explicitly distinguishing trajectories of adiposity (BMI and FMI) from fat-free mass (FFMI), moving beyond reliance on BMI alone.

The analyses reflect careful and rigorous work. The longitudinal design, large sample size, repeated objective measurements, and systematic consideration of confounding factors provide a strong foundation for the research questions addressed. The manuscript is well structured, written in clear and accessible English, and the results are presented in a transparent and coherent manner.

The comments below are offered in a constructive spirit, with the aim of strengthening transparency, conceptual clarity, and interpretability, rather than raising concerns about the validity of the study.

1. Handling of implausible values and outliers The manuscript does not explicitly describe whether implausible anthropometric or body composition values were identified and excluded, or how extreme values were handled prior to trajectory modelling. As latent class growth analysis can be sensitive to outliers—particularly in defining high trajectory groups—it would be helpful for reproducibility to briefly clarify whether the analyses relied exclusively on standard Millennium Cohort Study data cleaning procedures or whether any additional checks were performed.

2. Interpretation of FFMI in relation to body dissatisfaction The use of FFMI as a proxy for muscularity is appropriate and consistent with current epidemiological and clinical practice. There are no non-invasive methods that isolate muscle mass alone at scale, and FFMI remains the most widely accepted operational measure of fat-free mass. The observed associations between higher FFMI trajectories and greater body dissatisfaction should therefore be understood as an interpretative finding rather than a measurement limitation. Explicitly acknowledging the heterogeneous physiological and social meanings captured by FFMI during childhood and adolescence would further strengthen the discussion.

3. Hypotheses and observed sex differences The absence of sex differences for adiposity trajectories, alongside the positive association between higher FFMI trajectories and body dissatisfaction in both sexes, represents an important finding. Further reflection on evolving sociocultural pressures and contemporary body ideals could enrich the interpretation.

4. Outcome measure and interpretation of body dissatisfaction Body dissatisfaction is assessed using a validated single-item measure capturing global appearance-related dissatisfaction. While appropriate for cohort studies, this measure does not distinguish between specific dimensions of body image. Gently acknowledging this limitation in the Discussion or Conclusions would add conceptual nuance and better align interpretation with the scope of the measure.

5. Reporting guidelines and transparency Although adherence to reporting guidelines is not required by PLOS ONE, structured frameworks such as STROBE have been increasingly encouraged to enhance transparency and reproducibility. Optional reference to STROBE, and consideration of RECORD and GRoLTS guidelines, could further strengthen the manuscript.

Tables and consistency check A careful review of the tables indicated that they are generally internally consistent. One minor inconsistency was identified between the text and Table 3 for girls in the moderate FFMI trajectory, where the confidence interval appears to be a typographical error in the text. Aside from this, no duplicated values, incorrect totals, or inconsistencies were observed.

Overall, this is a carefully conducted study, and these comments are offered with the intention of further strengthening an already solid and valuable contribution.
