# Supplementary material for: Associations of childhood body composition trajectories and adolescent body dissatisfaction: Longitudinal evidence from the UK Millennium Cohort Study
Source: PLoS One. 2026 Oct 14;21(10):e0339555. doi: 10.1371/journal.pone.0339555 (PMC13667782; doi:10.1371/journal.pone.0339555)
Supplement: Supplementary file 2 [file pone.0339555.s001.docx]

# Supplemental information

# Methods

## Confounding variables

The following confounding variables were reported by the cohort member’s parent around 9 months of age.

Child characteristics, based on evidence of sex and ethnic differences in BMI and body dissatisfaction [1,2]:

- **Child sex** assigned at birth (Male or Female).
- **Child ethnicity**, according to Office for National Statistics 6-group classification (White; Mixed; Indian; Pakistani or Bangladeshi; Black or Black British; Other, including Chinese and Arab). We selected the 6-group classification due to observed differences in mean BMI between children of Indian and of Pakistani and Bangladeshi ethnicity.

Family socioeconomic position indicators, based on evidence that overweight and mental health difficulties are more prevalent in more disadvantaged children [1,3]:

- **Family income** was measured using OECD equivalised income quintiles, which account for household size and composition. This variable is derived by the Millennium Cohort Study team.
- **Highest educational qualification of parent(s) in the household** was measured as NVQ Level equivalents (None or overseas qualifications; NVQ Level 1, equivalent to GCSEs graded D or lower; NVQ Level 2, equivalent to GCSEs graded C or above; NVQ Level 3, equivalent to A Levels; NVQ Level 4, equivalent to an undergraduate degree; NVQ Level 5, equivalent to a higher degree). In order to include the father’s educational qualifications where available, we used the highest level of the available data.
- **Highest occupational class of the parent(s) in the household** was measured using the National Statistics Socio-economic classification, as five classes (Not in work; Routine or semi-routine; Lower supervisory or technical; Small employers or self-employed; Intermediate occupations; Managerial or professional). In order to include the father’s occupational class where available, we used the highest level of the available data.

Perinatal factors, based on evidence of their associations with child BMI [1] and eating disorders [4]:

- **Child birthweight** was measured in kilograms, with any reported in pounds and ounces converted. A continuous measure was used in regression analysis. For descriptive purposes only, a binary variable was generated to indicate whether the cohort member’s birthweight was classified as low birthweight (less than 2.5kg) [5].
- **Maternal pre-pregnancy BMI** was calculated from self-reported height and weight. A continuous measure was used in regression analysis, but for descriptive purposes only a categorical variable was generated to indicate whether the BMI was classified as under 25kg/m^2^ (not overweight), 25-29.9kg/m^2^ (overweight), or above 30kg/m^2^ (obese) [6].
- **Breastfeeding status** was assessed with the question “Did you ever try to breastfeed [your child]?” with the options Yes or No.
- **Cigarette use in pregnancy** was assessed using a series of questions capturing how many cigarettes were smoked per day prior to the pregnancy, whether this changed during pregnancy, and the number smoked per day after the change was made. A binary variable was generated to capture those who reported that they had not smoked during pregnancy (coded Never), or those who had continued to smoke, reduced, or quit during the pregnancy (coded Any).
- **Alcohol use in pregnancy** was assessed with a single question, “Thinking back to when you were pregnant, which of these best describes how often you usual drank then?”. A binary variable was generated to capture those who responded with a frequency from “Less than once a month” up to the most frequent “Everyday” (coded Any), or those responding Never (coded Never).

When the cohort member was approximately 3 years old, their main parent (usually their mother) completed the following psychological and child behavioural inventories:

Maternal psychological distress, based on evidence of associations with child BMI and depression [7,8]:

- **Maternal psychological distress** was assessed using the Kessler K6 scale, which is a validated tool to measure frequency of symptoms of psychological distress using six questions scored from 0 (none of the time) to 4 (all of the time) [9]. Scores for the six items were summed (range 0-24), with higher scores indicating greater levels of psychological distress. We used the continuous score in regression analyses. For descriptive purposes only, we derived a binary variable to indicate whether the Kessler score was interpreted as low (<5), moderate (5-12) or severe (≥13) levels of psychological distress using validated thresholds [10].

Child socioemotional difficulties, due to associations with BMI and body dissatisfaction [11,12]:

- **Child socioemotional** **difficulties** were measured using the Strengths and Difficulties Questionnaire. This is a well-validated 25-item behavioural screening questionnaire measuring emotional symptoms, conduct problems, hyperactivity/inattention, peer relationship problems, and prosocial behaviour [13]. Items are reported on a 3-point scale (0 = Not true; 1 = Somewhat true; 2 = Very true), and responses, excluding prosocial behaviour questions, are summed to give a total score of difficulties (range 0-40), where higher scores indicate more difficulties and increased likelihood of being diagnosed with a psychiatric disorder [14]. We used this continuous total score in regression analysis but used established thresholds to derive a categorical variable in descriptive analysis. These were: close to average (≤13); slightly raised (14-16); high (17-19); very high (≥20), as per SDQ scoring guidelines, see [15].

**SFig 1**. Simplified directed acyclic graph for the association between childhood body composition trajectory and adolescent body dissatisfaction.

## Latent Class Growth Analysis

### Model specification

Latent class growth analysis (LCGA) was performed in Stata MP version 17, using the *traj* package. For each measure of body composition, censored normal distribution models were estimated where each measure of body composition data up to age 14 years was the dependent variable and age in years was the independent variable. Beginning with a two-class model and increasing the number of classes, all combinations of polynomial shapes were modelled. Polynomial shapes for BMI models could be intercept only, linear, quadratic, or cubic. In LCGA of FMI and FFMI, the most complex polynomial shape that could be modelled was quadratic, as data for these measures were only available at three time points.

The *traj* package uses maximum likelihood estimation to account for missing body composition data, assuming that data are missing at random; therefore models were specified to be performed on a sample with at least one valid measurement of the body composition measure in each case.

### Model comparison, evaluation and visualisation of trajectories

The optimal number of classes and their polynomial shapes was chosen based on multiple metrics including goodness of fit (Akaike Information Criterion (AIC)) and neatness of classification (Entropy Index). For AIC, lower numbers represent improved fit for the data, while entropy indices closer to 1.0 represent more accurate class assignment. These were considered in tandem with each model’s ability to identify distinct and meaningful patterns in the data, while maintaining group sample sizes sufficient for subsequent analyses.

Estimated values and their 95% confidence intervals were exported to Excel, along with observed values for each class. To assess whether models were identifying distinct patterns in the data, estimated BMI values were plotted as line graphs alongside International Obesity Task Force cut off values [16]. This provided context to the trajectory values. However, as there are no accepted international cut off values for FMI and FFMI, we took the 5^th^, 85^th^ and 95^th^ centile values for both body composition measures from the MCS sample itself and plotted alongside the model estimates to provide some further meaning. These centiles were selected as they correspond to World Health Organisation and United States BMI centiles for overweight (85^th^ centile) [17,18]; they are not intended to categorise body composition trajectories but provide some context.

After selecting the optimal trajectory model, participants were assigned to their most likely class, thus creating a categorical variable to represent their trajectory group for each body composition measure. This is the most commonly used three-step method of latent class analysis, also known as Modal Standard [19].

In the supplemental results below, we present information about missing data and the distribution of observed data across waves. Also in the supplemental results, we report the AIC and Entropy index results for increasing numbers of classes below, based on the optimal polynomial shapes and the mean trajectories for each model graphically. We also show AIC and Entropy results for different polynomial shapes among the optimal number of classes.

## Multiple imputation

Auxiliary variables were raw measures of child BMI, FMI, FFMI and SDQ scores at all waves, body dissatisfaction score at age 11, and child-reported depressive symptoms at age 14 using the short Moods and Feelings Questionnaire.

# Results

## Body composition trajectories

**Table S1.** Means of BMI, FMI and FFMI measures

| Mean (SD) |  | Age 3 | Age 5 | Age 7 | Age 11 | Age 14 |
| --- | --- | --- | --- | --- | --- | --- |
| Boys | |  |  |  |  |  |
| *BMI* | N | 6924 | 6590 | 6032 | 5691 | 4917 |
|  | Missing % | 7.4 | 11.8 | 19.3 | 23.8 | 34.2 |
|  | Mean (SD) | 17.02 (2.2) | 16.39 (1.9) | 16.60 (2.3) | 19.01 (3.5) | 20.93 (4.0) |
|  | Underweight | 4.7 | 5.3 | 6.1 | 5.7 | 7.6 |
| *%* | Healthy | 72.0 | 75.4 | 75.8 | 68.7 | 67.1 |
|  | Overweight | 17.6 | 14.0 | 12.6 | 19.6 | 18.1 |
|  | Very overweight | 5.7 | 5.3 | 5.4 | 6.0 | 7.2 |
|  | N | - | - | 5943 | 5621 | 4844 |
|  | Missing % |  |  | 20.5 | 24.8 | 35.2 |
| *FMI* | Mean (SD) |  |  | 3.44 (1.5) | 4.07 (2.5) | 3.80 (2.7) |
| *FFMI* | Mean (SD) |  |  | 13.16 (1.1) | 14.94 (1.5) | 17.12 (1.9) |
| Girls | |  |  |  |  |  |
| *BMI* | N | 6778 | 6376 | 5950 | 5590 | 4789 |
|  | Missing (%) | 5.8 | 11.4 | 17.3 | 22.3 | 33.5 |
|  | Mean (SD) | 16.70 (1.9) | 16.30 (1.9) | 16.69 (2.4) | 19.45 (3.7) | 21.94 (4.1) |
|  | Underweight | 5.2 | 4.4 | 6.2 | 7.3 | 6.2 |
| *%* | Healthy | 70.5 | 71.9 | 70.6 | 63.2 | 66.3 |
|  | Overweight | 18.5 | 17.7 | 16.5 | 22.5 | 20.2 |
|  | Very overweight | 5.8 | 6.0 | 6.7 | 7.0 | 7.2 |
|  | N | - | - | 5867 | 5536 | 4736 |
|  | Missing (%) |  |  | 18.5 | 23.1 | 34.2 |
| *FMI* | Mean (SD) |  |  | 3.82 (1.6) | 5.04 (2.5) | 6.20 (2.9) |
| *FFMI* | Mean (SD) |  |  | 12.87 (1.0) | 14.39 (1.4) | 15.73 (1.4) |

**Table S2.** Sex-specific means of BMI within BMI, FMI and FFMI trajectory groups.

| Boys mean | Age 3 | Age 5 | Age 7 | Age 11 | Age 14 |
| --- | --- | --- | --- | --- | --- |
| BMI Trajectory |  |  |  |  |  |
| Low BMI | 16.5 | 15.8 | 15.6 | 17.2 | 18.7 |
| Moderate BMI | 17.8 | 17.3 | 17.9 | 21.9 | 24.2 |
| High BMI | 19.9 | 20.3 | 22.4 | 27.7 | 31.0 |
| FMI Trajectory |  |  |  |  |  |
| Low FMI | 16.8 | 16.8 | 15.9 | 17.7 | 19.4 |
| Moderate FMI | 17.6 | 17.6 | 18.5 | 23.0 | 26.0 |
| High FMI | 19.0 | 19.9 | 22.6 | 28.3 | 32.1 |
| FFMI Trajectory |  |  |  |  |  |
| Low FFMI | 16.2 | 15.3 | 15.0 | 16.3 | 17.8 |
| Moderate FFMI | 17.2 | 16.5 | 16.8 | 19.3 | 21.1 |
| High FFMI | 18.3 | 18.3 | 19.7 | 24.2 | 26.8 |
| Girls mean | **Age 3** | **Age 5** | **Age 7** | **Age 11** | **Age 14** |
| BMI Trajectory |  |  |  |  |  |
| Low BMI | 16.2 | 15.5 | 15.4 | 17.2 | 19.4 |
| Moderate BMI | 17.3 | 17.1 | 17.9 | 21.9 | 24.5 |
| High BMI | 19.3 | 19.9 | 22.0 | 27.6 | 31.7 |
| FMI Trajectory |  |  |  |  |  |
| Low FMI | 16.3 | 15.6 | 15.4 | 17.0 | 19.2 |
| Moderate FMI | 17.3 | 16.9 | 17.6 | 21.4 | 24.1 |
| High FMI | 18.5 | 19.3 | 21.6 | 27.3 | 31.6 |
| FFMI Trajectory |  |  |  |  |  |
| Low FFMI | 13.1 | 15.4 | 15.3 | 17.1 | 19.5 |
| Moderate FFMI | 17.2 | 17.0 | 17.9 | 21.9 | 24.7 |
| High FFMI | 18.8 | 19.9 | 22.4 | 28.8 | 33.5 |

**Table S3.** Summary of Latent Class Growth Analysis modelling results

| Model and Number of Classes | AIC | Entropy |
| --- | --- | --- |
| BMI modelling |  |  |
| 2 | -137700.98 | 0.831 |
| 3 | -133965.65 | 0.786 |
| 4 | -132343.57 | 0.728 |
| 5 | -130978.68 | 0.763 |
| FMI modelling |  |  |
| 2 | -67272.92 | 0.872 |
| 3 | -63993.01 | 0.841 |
| 4 | -62371.31 | 0.745 |
| 5 | -61456.30 | 0.696 |
| FFMI modelling |  |  |
| 2 | -53907.96 | 0.722 |
| 3 | -52132.05 | 0.701 |
| 4 | -51311.81 | 0.702 |
| 5 | -51120.97 | 0.697 |

**Figure S2.** Plots showing estimated mean trajectories for each model

(A) **BMI**

|  | Base | Under | Healthy | Over | Obese |
| --- | --- | --- | --- | --- | --- |
| 3 | 14 | 0.83 | 3.02 | 1.65 | 14.5 |
| 4 | 14 | 0.51 | 3.01 | 1.71 | 14.77 |
| 5 | 14 | 0.26 | 3.13 | 1.88 | 14.73 |
| 6 | 14 | 0.06 | 3.46 | 2.24 | 14.24 |
| 7 | 14 | 0 | 3.88 | 2.71 | 13.41 |
| 8 | 14 | 0.13 | 4.28 | 3.15 | 12.44 |
| 9 | 14 | 0.36 | 4.71 | 3.64 | 11.29 |
| 10 | 14 | 0.63 | 5.17 | 4.16 | 10.04 |
| 11 | 14 | 0.96 | 5.55 | 4.56 | 8.93 |
| 12 | 14 | 1.36 | 5.84 | 4.82 | 7.98 |
| 13 | 14 | 1.84 | 6.05 | 4.98 | 7.13 |
| 14 | 14 | 2.39 | 6.21 | 5.04 | 6.36 |
| 15 | 14 | 2.98 | 6.3 | 5.04 | 5.68 |

|  |  |  |  |
| --- | --- | --- | --- |
| 3.14 | 15.45 | 16.29 | 18.37 |
| 5.22 | 15.98 | 17.67 | 20.7 |
| 7.24 | 16.5 | 19.01 | 22.97 |
| 11.17 | 17.5 | 21.62 | 27.38 |
| 14.26 | 18.29 | 23.67 | 30.85 |

BMI

|  | Base | Under | Healthy | Over | Obese |
| --- | --- | --- | --- | --- | --- |
| 3 | 14 | 0.83 | 3.02 | 1.65 | 14.5 |
| 4 | 14 | 0.51 | 3.01 | 1.71 | 14.77 |
| 5 | 14 | 0.26 | 3.13 | 1.88 | 14.73 |
| 6 | 14 | 0.06 | 3.46 | 2.24 | 14.24 |
| 7 | 14 | 0 | 3.88 | 2.71 | 13.41 |
| 8 | 14 | 0.13 | 4.28 | 3.15 | 12.44 |
| 9 | 14 | 0.36 | 4.71 | 3.64 | 11.29 |
| 10 | 14 | 0.63 | 5.17 | 4.16 | 10.04 |
| 11 | 14 | 0.96 | 5.55 | 4.56 | 8.93 |
| 12 | 14 | 1.36 | 5.84 | 4.82 | 7.98 |
| 13 | 14 | 1.84 | 6.05 | 4.98 | 7.13 |
| 14 | 14 | 2.39 | 6.21 | 5.04 | 6.36 |
| 15 | 14 | 2.98 | 6.3 | 5.04 | 5.68 |

|  | Low |  |
| --- | --- | --- |
| 3.14 | 15.58 | 17.16 |
| 5.22 | 16.24 | 19.03 |
| 7.24 | 16.88 | 20.83 |
| 11.17 | 18.13 | 24.36 |
| 14.26 | 19.1 | 27.14 |

BMI

|  | Base | Under | Healthy | Over | Obese |
| --- | --- | --- | --- | --- | --- |
| 3 | 14 | 0.83 | 3.02 | 1.65 | 14.5 |
| 4 | 14 | 0.51 | 3.01 | 1.71 | 14.77 |
| 5 | 14 | 0.26 | 3.13 | 1.88 | 14.73 |
| 6 | 14 | 0.06 | 3.46 | 2.24 | 14.24 |
| 7 | 14 | 0 | 3.88 | 2.71 | 13.41 |
| 8 | 14 | 0.13 | 4.28 | 3.15 | 12.44 |
| 9 | 14 | 0.36 | 4.71 | 3.64 | 11.29 |
| 10 | 14 | 0.63 | 5.17 | 4.16 | 10.04 |
| 11 | 14 | 0.96 | 5.55 | 4.56 | 8.93 |
| 12 | 14 | 1.36 | 5.84 | 4.82 | 7.98 |
| 13 | 14 | 1.84 | 6.05 | 4.98 | 7.13 |
| 14 | 14 | 2.39 | 6.21 | 5.04 | 6.36 |
| 15 | 14 | 2.98 | 6.3 | 5.04 | 5.68 |

|  |  | 16.73 18.67 20.56 24.24 27.13 |  |  |
| --- | --- | --- | --- | --- |
| 3.14 | 15.31 | 16.73 | 16.15 | 19.62 |
| 5.22 | 15.77 | 18.67 | 17.2 | 22.17 |
| 7.24 | 16.22 | 20.56 | 18.22 | 24.64 |
| 11.17 | 17.1 | 24.24 | 20.21 | 29.46 |
| 14.26 | 17.78 | 27.13 | 21.78 | 33.24 |

|  | Base | Under | Healthy | Over | Obese |
| --- | --- | --- | --- | --- | --- |
| 3 | 14 | 0.83 | 3.02 | 1.65 | 14.5 |
| 4 | 14 | 0.51 | 3.01 | 1.71 | 14.77 |
| 5 | 14 | 0.26 | 3.13 | 1.88 | 14.73 |
| 6 | 14 | 0.06 | 3.46 | 2.24 | 14.24 |
| 7 | 14 | 0 | 3.88 | 2.71 | 13.41 |
| 8 | 14 | 0.13 | 4.28 | 3.15 | 12.44 |
| 9 | 14 | 0.36 | 4.71 | 3.64 | 11.29 |
| 10 | 14 | 0.63 | 5.17 | 4.16 | 10.04 |
| 11 | 14 | 0.96 | 5.55 | 4.56 | 8.93 |
| 12 | 14 | 1.36 | 5.84 | 4.82 | 7.98 |
| 13 | 14 | 1.84 | 6.05 | 4.98 | 7.13 |
| 14 | 14 | 2.39 | 6.21 | 5.04 | 6.36 |
| 15 | 14 | 2.98 | 6.3 | 5.04 | 5.68 |

|  |  |  |  |  |  |
| --- | --- | --- | --- | --- | --- |
| 3.14 | 16.03 | 16.7 | 15.29 | 25.73 | 18.97 |
| 5.22 | 17.1 | 18.62 | 15.75 | 23.92 | 21.67 |
| 7.24 | 18.14 | 20.48 | 16.19 | 22.17 | 24.28 |
| 11.17 | 20.16 | 24.11 | 17.06 | 18.74 | 29.38 |
| 14.26 | 21.75 | 26.97 | 17.75 | 16.06 | 33.38 |

(B) **FMI**

FMI

|  | 1 | 2 | 5th | 85th | 95th |
| --- | --- | --- | --- | --- | --- |
| 7 | 3.2 | 5.84 | 2.05 | 4.82 | 6.69 |
| 11 | 3.6 | 8.25 | 1.84 | 7.08 | 9.51 |
| 14 | 3.93 | 10.14 | 1.5 | 7.84 | 10.94 |

|  | 4.46 | 2 | 5th | 85th | 95th | 1 |
| --- | --- | --- | --- | --- | --- | --- |
| 7 | 3 | 4.46 | 2.05 | 4.82 | 6.69 | 7.42 |
| 11 | 3.24 | 6.2 | 1.84 | 7.08 | 9.51 | 10.62 |
| 14 | 3.42 | 7.57 | 1.5 | 7.84 | 10.94 | 13.13 |

FMI

| Age | 3 | 2 | 5th | 85th | 95th | 1 | 4 |
| --- | --- | --- | --- | --- | --- | --- | --- |
| 7 | 2.89 | 3.92 | 2.05 | 4.82 | 6.69 | 5.69 | 8.87 |
| 11 | 2.99 | 5.25 | 1.84 | 7.08 | 9.51 | 8.2 | 12.61 |
| 14 | 3.07 | 6.3 | 1.5 | 7.84 | 10.94 | 10.16 | 15.54 |

| Age | 2.81 2.8 2.79 | 2 | 5th | 85th | 95th | 4.84 6.84 8.41 | 4 | 5+'FMI plot details'!$F$21 |
| --- | --- | --- | --- | --- | --- | --- | --- | --- |
| 7 | 2.81 | 3.57 | 2.05 | 4.82 | 6.69 | 4.84 | 6.61 | 9.81 |
| 11 | 2.8 | 4.6 | 1.84 | 7.08 | 9.51 | 6.84 | 9.93 | 13.96 |
| 14 | 2.79 | 5.42 | 1.5 | 7.84 | 10.94 | 8.41 | 12 | 17.22 |

(C) **FFMI**

FFMI

|  |  | 2 | 5th | 85th | 95th |
| --- | --- | --- | --- | --- | --- |
| 7 | 12.57 | 13.88 | 11.54 | 13.98 | 14.8 |
| 11 | 14.22 | 16.42 | 12.57 | 16.09 | 17.14 |
| 14 | 15.51 | 18.42 | 13.94 | 18.25 | 19.69 |

|  | 1 | 2 | 5th | 85th | 95th | 3 |
| --- | --- | --- | --- | --- | --- | --- |
| 7 | 12.29 | 13.26 | 11.54 | 13.98 | 14.8 | 14.47 |
| 11 | 13.79 | 15.35 | 12.57 | 16.09 | 17.14 | 17.43 |
| 14 | 14.96 | 16.99 | 13.94 | 18.25 | 19.69 | 19.75 |

FFMI

| Age | 1 | 2 | 5th | 85th | 95th | 3 | 4 |
| --- | --- | --- | --- | --- | --- | --- | --- |
| 7 | 12.07 | 12.92 | 11.54 | 13.98 | 14.8 | 13.79 | 15.15 |
| 11 | 13.46 | 14.77 | 12.57 | 16.09 | 17.14 | 16.31 | 18.43 |
| 14 | 14.55 | 16.22 | 13.94 | 18.25 | 19.69 | 18.29 | 21.01 |

| Age | 1 | 2 | 5th | 85th | 95th | 3 | 4 | 5 |
| --- | --- | --- | --- | --- | --- | --- | --- | --- |
| 7 | 12.08 | 12.94 | 11.54 | 13.98 | 14.8 | 13.8 | 13.97 | 15.15 |
| 11 | 13.49 | 14.79 | 12.57 | 16.09 | 17.14 | 16.33 | 9.64 | 18.44 |
| 14 | 14.59 | 16.25 | 13.94 | 18.25 | 19.69 | 18.31 | 6.25 | 21.02 |

**Table S4.** Results of trajectory modelling using different polynomial shapes

(A) **BMI**

| **Number of Classes** | **Polynomials** | **Akaike Information Criterion** | **Entropy Index** | **Group Membership %** |
| --- | --- | --- | --- | --- |
| 3 | 0  0  0 | -152649.55 | 0.834 | 81.2  16.4  2.4 |
| **3** | **1**  **1**  **1** | **-133965.65** | **0.786** | **63.0**  **30.0**  **7.0** |
| 3 | 1  1  2 | -133722.76 | 0.770 | 60.7  30.6  8.7 |
| 3 | 1  1  3 | -133548.29 | 0.762 | 59.5  31.0  9.5 |
| 3 | 1  2  1 | -132312.8 | 0.749 | 52.6  38.6  8.7 |
| 3 | 1  2  2 | -132040.07 | 0.735 | 49.5  40.1  10.4 |
| 3 | 1  2  3 | -131837.78 | 0.727 | 47.7  41.0  11.2 |
| 3 | 1  3  1 | -131824.4 | 0.761 | 53.3  38.4  8.3 |
| 3 | 1  3  2 | -131582.87 | 0.749 | 50.8  39.5  9.7 |
| 3 | 1  3  3 | -131401.42 | 0.741 | 49.2  20.3  10.5 |
| 3 | 2  1  1 | -132312.8 | 0.749 | 38.6  52.6  8.7 |
| 3 | 2  1  2 | -132040.07 | 0.735 | 40.1  49.5  10.4 |
| 3 | 2  1  3 | -135303.08 | 0.882 | 23.4  76.1  0.5 |
| 3 | 2  2  1 | -129823.04 | 0.802 | 61.7  31.4  6.9 |
| 3 | 2  2  2 | -129616.51 | 0.795 | 60.4  31.8  7.8 |
| 3 | 2  2  3 | -132502.84 | 0.899 | 77.6  21.8  0.5 |
| 3 | 2  3  1 | -129315.13 | 0.805 | 61.3  32.0  6.7 |
| 3 | 2  3  2 | -129458.34 | 0.790 | 59.5  8.4  32.1 |
| 3 | 2  3  3 | -128980.11 | 0.794 | 59.2  32.7  8.1 |
| 3 | 3  1  1 | -131824.4 | 0.761 | 38.4  53.3  8.3 |
| 3 | 3  1  2 | -130794.49 | 0.823 | 67.6  25.8  6.6 |
| 3 | 3  1  3 | -131401.42 | 0.741 | 40.3  49.2  10.5 |
| 3 | 3  2  1 | -129735.77 | 0.804 | 62.3  30.9  6.8 |
| 3 | 3  2  2 | -129533.71 | 0.797 | 61.1  31.3  7.6 |
| 3 | 3  2  3 | -129378.8 | 0.792 | 60.2  31.6  8.2 |
| 3 | 3  3  1 | -129242.11 | 0.807 | 61.8  31.5  6.6 |
| 3 | 3  3  2 | -129051.9 | 0.802 | 60.9  31.8  7.3 |
| 3 | 3  3  3 | -128914.83 | 0.797 | 59.9  32.2  7.9 |

(B) **FMI**

| **Number of Classes** | **Polynomials** | **Akaike Information Criterion** | **Entropy Index** | **Group Membership %** |
| --- | --- | --- | --- | --- |
| 3 | 0  0  0 | -68700.65 | 0.863 | 75.9  20.4  3.7 |
| **3** | **1**  **1**  **1** | **-63993.01** | **0.841** | **26.9**  **66.7**  **6.4** |
| 3 | 1  1  2 | -63972.24 | 0.840 | 66.5  27.0  6.5 |
| 3 | 1  2  1 | -63916.66 | 0.844 | 66.9  26.9  6.2 |
| 3 | 1  2  2 | -63897.1 | 0.844 | 66.7  27.0  6.3 |
| 3 | 2  1  1 | -63993.29 | 0.841 | 66.7  26.9  6.4 |
| 3 | 2  1  2 | -63891.1 | 0.844 | 27.0  66.7  6.3 |
| 3 | 2  2  1 | -63947.27 | 0.845 | 26.9  66.9  6.2 |
| 3 | 2  2  2 | -63897.72 | 0.844 | 66.7  27.0  6.3 |

(C) **FFMI**

| **Number of Classes** | **Polynomials** | **Akaike Information Criterion** | **Entropy Index** | **Group Membership %** |
| --- | --- | --- | --- | --- |
| 3 | 0  0  0 | -68448.03 | 0.400 | 67.8  26.7  5.5 |
| **3** | **1**  **1**  **1** | **-52132.05** | **0.701** | **44.9**  **44.9**  **10.1** |
| 3 | 1  1  2 | -52085.95 | 0.599 | 44.3  45.1  10.6 |
| 3 | 1  2  1 | -53666.27 | 0.823 | 70.4  29.5  0.2 |
| 3 | 1  2  2 | -51969.71 | 0.698 | 42.9  46.2  10.8 |
| 3 | 2  1  1 | -52011.56 | 0.700 | 46.0  43.6  10.4 |
| 3 | 2  1  2 | -51854.58 | 0.708 | 45.2  9.9  44.9 |
| 3 | 2  2  1 | -51854.58 | 0.708 | 45.2  44.9  9.9 |
| 3 | 2  2  2 | -51813.22 | 0.706 | 44.6  45.0  10.4 |

## Missing data

**Table S5.** Comparison of sample characteristics among those with valid exposure data and those with complete data on exposure, outcome and confounders.

| Variable | **Valid Exposure Data**  **N (%)** | **Complete Data**  **N (%)** |
| --- | --- | --- |
| N | 13025 | 7933 |
| **Sex at birth** |  |  |
| Male | 6612 (50.8) | 3904 (49.2) |
| Female | 6413 (49.2) | 4029 (50.8) |
| *Missing^a^* | 0 | - |
| **Ethnicity** |  |  |
| White | 10968 (84.4) | 7157 (90.2) |
| Mixed | 346 (2.7) | 197 (2.5) |
| Indian | 312 (2.4) | 130 (1.6) |
| Pakistani or Bangladeshi | 828 (6.4) | 249 (3.1) |
| Black or Black British | 383 (2.9) | 145 (1.8) |
| Other, including Chinese or Arab | 160 (1.2) | 55 (0.7) |
| *Missing* | 28 (0.2) | - |
| **Family income at 9 months** |  |  |
| Most disadvantaged | 2804 (21.6) | 1250 (15.8) |
| Second | 2786 (21.4) | 1506 (19.0) |
| Third | 2526 (19.4) | 1626 (20.5) |
| Fourth | 2521 (19.4) | 1801 (22.7) |
| Least disadvantaged | 2355 (18.1) | 1750 (22.1) |
| *Missing* | 33 (0.3) | - |
| **Highest parent education attainment at 9 months** |  |  |
| None | 1403 (10.8) | 469 (5.9) |
| NVQ Level 1 | 775 (5.9) | 307 (4.7) |
| NVQ Level 2 | 3199 (24.6) | 1817 (22.9) |
| NVQ Level 3 | 2151 (16.5) | 1370 (17.3) |
| NVQ Level 4 | 4593 (35.3) | 3257 (41.1) |
| NVQ Level 5 | 896 (6.9) | 650 (8.2) |
| *Missing* | 8 (0.1) | - |
| **Highest parent occupational class at 9 months** |  |  |
| Not in work | 2924 (22.5) | 1309 (16.5) |
| Routine or semi-routine | 1900 (14.6) | 1021 (12.9) |
| Lower supervisory or technical | 1043 (8.0) | 612 (7.7) |
| Small employers or self-employed | 863 (6.6) | 515 (6.5) |
| Intermediate occupations | 1199 (9.2) | 787 (9.9) |
| Managerial or professional | 5050 (38.9) | 3689 (46.5) |
| *Missing* | 46 (0.4) | - |
| **Birthweight** |  |  |
| Low birthweight (≤2.5kg) | 890 (6.9) | 443 (5.6) |
| Birthweight >2.5kg | 12106 (93.1) | 7490 (94.4) |
| *Missing* | 29 (0.2) | - |
| **Maternal pre-pregnancy BMI** |  |  |
| BMI <25 | 8416 (70.3) | 5614 (70.8) |
| BMI 25 – 29.9 | 2465 (20.6) | 1621 (20.4) |
| BMI ≥30 | 1083 (9.1) | 698 (8.8) |
| *Missing* | 1061 (8.2) | - |
| **Breastfed** |  |  |
| No | 3870 (29.8) | 2102 (26.5) |
| Yes | 9135 (70.2) | 58.31 (73.5) |
| *Missing* | 20 (0.2) | - |
| **Cigarette use in pregnancy** |  |  |
| None | 9455 (72.6) | 5839 (73.6) |
| Any | 3564 (27.4) | 2174 (27.4) |
| *Missing* | 6 (0.1) | - |
| **Alcohol use in pregnancy** |  |  |
| None | 9020 (69.4) | 5202 (65.6) |
| Any | 3982 (30.6) | 2731 (34.4) |
| *Missing* | 23 (0.2) | - |
| **Maternal psychological distress score at 3 years** |  |  |
| Low | 8591 (75.0) | 6068 (76.5) |
| Moderate | 2472 (21.6) | 1631 (20.6) |
| High | 388 (3.4) | 234 (2.9) |
| *Missing* | 1574 (12.1) | - |
| **Child total SDQ score at 3 years** |  |  |
| Close to average | 9594 (79.0) | 6525 (82.2) |
| Slightly raised | 1276 (10.5) | 791 (9.6) |
| High | 678 (5.6) | 371 (4.7) |
| Very high | 593 (4.9) | 276 (3.5) |
| *Missing* | 884 (6.8) | - |
| **Body dissatisfaction at 14 years** |  |  |
| Mean (SD) | 2.21 (1.6) | 2.24 (1.6) |
| *Missing* | 3151 (24.2) | - |

a Proportions of non-missing data add up to 100% for comparisons to be made across samples.

**Table S6**. Proportions of missing and available outcome data at 14 among sample characteristics in participants with valid body composition trajectory data (n=13,025).

| Variable | **Missing body dissatisfaction score at 14 years** | |
| --- | --- | --- |
|  | No  (n= 9874, 75.8%) | Yes  (n= 3151, 24.2%) |
| **Sex at birth** |  |  |
| Male | 73.7 | 26.3 |
| Female | 77.9 | 22.1 |
| **Ethnicity** |  |  |
| White | 75.3 | 24.7 |
| Mixed | 72.5 | 27.5 |
| Indian | 82.1 | 17.9 |
| Pakistani or Bangladeshi | 82.3 | 17.7 |
| Black or Black British | 72.1 | 27.9 |
| Other, including Chinese and Arab | 82.5 | 17.5 |
| **Family income at 9 months** |  |  |
| Most disadvantaged | 67.4 | 32.6 |
| Second | 72.8 | 27.2 |
| Third | 76.2 | 23.8 |
| Fourth | 81.2 | 18.8 |
| Least disadvantaged | 83.4 | 16.6 |
| **Highest parental education level at 9 months** |  |  |
| None or overseas | 68.1 | 31.9 |
| NVQ Level 1 | 67.1 | 32.9 |
| NVQ Level 2 | 70.5 | 29.5 |
| NVQ Level 3 | 73.8 | 26.2 |
| NVQ Level 4 | 82.0 | 18.0 |
| NVQ Level 5 | 87.5 | 12.5 |
| **Highest parental occupational class at 9 months** |  |  |
| Not in work | 67.0 | 33.0 |
| Routine or semi-routine | 71.9 | 28.1 |
| Lower supervisory or technical | 72.1 | 27.9 |
| Small employers or self-employed | 75.2 | 24.8 |
| Intermediate occupations | 76.6 | 23.4 |
| Managerial or professional | 83.0 | 17.0 |
| **Birthweight** |  |  |
| Low birthweight (≤2.5kg) | 73.8 | 26.2 |
| Birthweight >2.5kg | 76.0 | 24.0 |
| **Maternal pre-pregnancy BMI** |  |  |
| BMI <25 | 76.2 | 23.8 |
| BMI 25 – 29.9 | 76.0 | 24.0 |
| BMI ≥30 | 75.2 | 24.8 |
| **Breastfed** |  |  |
| No | 69.1 | 30.9 |
| Yes | 78.7 | 21.3 |
| **Cigarette use in pregnancy** |  |  |
| None | 76.9 | 23.1 |
| Any | 69.6 | 30.4 |
| **Alcohol use in pregnancy** |  |  |
| None | 75.1 | 24.9 |
| Any | 77.6 | 22.4 |
| **Maternal psychological distress score at 3 years** |  |  |
| Low | 76.9 | 23.1 |
| Moderate | 73.9 | 26.1 |
| High | 69.1 | 30.9 |
| **Child total SDQ score at 3 years** |  |  |
| Close to average | 77.8 | 22.2 |
| Slightly raised | 71.0 | 29.0 |
| High | 69.3 | 30.7 |
| Very high | 64.4 | 35.6 |
| **BMI Trajectory** |  |  |
| Low | 74.1 | 25.9 |
| Moderate | 79.7 | 20.3 |
| High | 75.2 | 24.8 |
| **FMI Trajectory** |  |  |
| Low | 74.4 | 25.6 |
| Moderate | 80.3 | 19.7 |
| High | 72.6 | 27.4 |
| **FFMI Trajectory** |  |  |
| Low | 77.0 | 23.0 |
| Moderate | 74.1 | 25.9 |
| High | 78.5 | 21.4 |

## Sensitivity analyses

**Table S7.** Comparison of unadjusted results in a sample with valid BMI trajectory and the sample restricted to those with other trajectory data (collected from age 7 onward). Missing outcome data imputed, sampling weights applied.

| **BMI Trajectory** | *Mean difference in body dissatisfaction at age 14*  *(95% Confidence Interval)* | |
| --- | --- | --- |
|  | **Full sample**  **(n= 14,675)** | **Restricted sample**  **(n= 13,025)** |
| Low | ref | ref |
| Moderate | 0.41  (0.33, 0.48) | 0.41  (0.34, 0.49) |
| High | 0.86  (0.70, 1.01) | 0.87  (0.71, 1.02) |

**Table S8.** Results of univariable and multivariable linear regression analysis of the association between body composition trajectory and body dissatisfaction at age 14 in participants with complete data on exposures, outcomes and confounders (N=7933, sampling and attrition weights applied).

| **Model** | *Mean difference in body dissatisfaction at age 14 years (95% Confidence Interval)* | | | | | | | | |
| --- | --- | --- | --- | --- | --- | --- | --- | --- | --- |
|  | **BMI Trajectory** | | | **FMI Trajectory** | | | **FFMI Trajectory** | | |
|  | Low | Moderate | High | Low | Moderate | High | Low | Moderate | High |
| Model 0  (unadjusted) | ref | 0.40  (0.30, 0.49) | 0.93  (0.72, 1.15) | ref | 0.64  (0.53, 0.75) | 1.15  (0.92, 1.37) | ref | 0.05  (-0.05, 0.14) | 0.17  (0.02, 0.32) |
| Model 1  (0 + child characteristics^a^) | ref | 0.32  (0.23, 0.41) | 0.86  (0.65, 1.07) | ref | 0.40  (0.29, 0.51) | 0.93  (0.70, 1.15) | ref | 0.28  (0.19, 0.38) | 0.66  (0.51, 0.81) |
| Model 2  (0 + family SEP^b^) | ref | 0.38  (0.29, 0.47) | 0.89  (0.68, 1.10) | ref | 0.62  (0.51, 0.73) | 1.11  (0.88, 1.33) | ref | 0.04  (-0.06, 0.13) | 0.14  (-0.01, 0.28) |
| Model 3  (0 + perinatal factors^c^) | ref | 0.37  (0.27, 0.47) | 0.86  (0.63, 1.08) | ref | 0.61  (0.50, 0.72) | 1.10  (0.87, 1.33) | ref | 0.01  (-0.09, 0.11) | 0.07  (-0.09, 0.24) |
| Model 4  (0 + maternal psychological distress^d^) | ref | 0.39  (0.30, 0.49) | 0.92  (0.71, 1.14) | ref | 0.63  (0.52, 0.74) | 1.14  (0.91, 1.36) | ref | 0.04  (-0.05, 0.14) | 0.16  (0.01, 0.31) |
| Model 5  (0 + child socioemotional difficulties^e^) | ref | 0.39  (0.30, 0.49) | 0.91  (0.70, 1.13) | ref | 0.64  (0.53, 0.75) | 1.14  (0.91, 1.36) | ref | 0.04  (-0.06, 0.14) | 0.14  (-0.01, 0.29) |
| Model 6  (0 + all confounders in Models 1-5) | ref | 0.29  (0.19, 0.37) | 0.75  (0.54, 0.96) | ref | 0.34  (0.23, 0.45) | 0.82  (0.59, 1.05) | ref | 0.24  (0.14, 0.33) | 0.55  (0.39, 0.71) |
| Exposure × sex interaction  p value | ref | 0.135 | 0.821 | ref | 0.997 | 0.441 | ref | <0.001 | 0.012 |
| Mean difference in boys  (N=3904) | ref | - | - | ref |  |  | ref | 0.04  (-0.08, 0.17) | 0.40  (0.22, 0.58) |
| Mean difference in girls  (N=6413) | ref | - | - | ref |  |  | ref | 0.39  (0.25, 0.53) | 0.82  (0.45, 1.19) |

^a^ Child sex at birth, ethnicity. ^b^ Family income quintile, parental educational qualification level, parental occupational class. ^c^ Child birthweight, maternal pre-pregnancy BMI, breastfeeding, cigarette and alcohol use in pregnancy. ^d^ Maternal psychological distress score at 3 years old. ^e^ Child total SDQ score at 3 years old

**References**

1.	Kelly Y, Patalay P, Montgomery S, Sacker A. BMI Development and Early Adolescent Psychosocial Well-Being: UK Millennium Cohort Study. Pediatrics. 2016;138: e20160967. doi:10.1542/peds.2016-0967

2.	Quick V, Eisenberg ME, Bucchianeri MM, Neumark-Sztainer D. Prospective Predictors of Body Dissatisfaction in Young Adults: 10-year Longitudinal Findings. Emerg Adulthood. 2013;1: 271–282. doi:10.1177/2167696813485738

3.	Hazell M, Thornton E, Haghparast-Bidgoli H, Patalay P. Socio-economic inequalities in adolescent mental health in the UK: Multiple socio-economic indicators and reporter effects. SSM - Mental Health. 2022;2: 100176. doi:10.1016/j.ssmmh.2022.100176

4.	Goodman A, Heshmati A, Malki N, Koupil I. Associations between birth characteristics and eating disorders across the life course: findings from 2 million males and females born in Sweden, 1975-1998. Am J Epidemiol. 2014;179: 852–863. doi:10.1093/aje/kwt445

5.	Global nutrition targets 2025: low birth weight policy brief. [cited 14 Mar 2023]. Available: https://www.who.int/publications/i/item/WHO-NMH-NHD-14.5

6.	World Health Organization, editor. International statistical classification of diseases and related health problems. 10th revision, 2nd edition. Geneva: World Health Organization; 2004.

7.	Tommerup K, Lacey RE. Maternal and Paternal Distress in Early Childhood and Child Adiposity Trajectories: Evidence from the Millennium Cohort Study. Obesity (Silver Spring). 2021;29: 888–899. doi:10.1002/oby.23150

8.	Pearson RM, Evans J, Kounali D, Lewis G, Heron J, Ramchandani PG, et al. Maternal Depression During Pregnancy and the Postnatal Period: Risks and Possible Mechanisms for Offspring Depression at Age 18 Years. JAMA Psychiatry. 2013;70: 1312–1319. doi:10.1001/jamapsychiatry.2013.2163

9.	Kessler RC, Andrews G, Colpe LJ, Hiripi E, Mroczek DK, Normand S-LT, et al. Short screening scales to monitor population prevalences and trends in non-specific psychological distress. Psychological Medicine. 2002;32: 959–976. doi:10.1017/S0033291702006074

10.	Prochaska JJ, Sung H-Y, Max W, Shi Y, Ong M. Validity study of the K6 scale as a measure of moderate mental distress based on mental health treatment need and utilization. Int J Methods Psychiatr Res. 2012;21: 88–97. doi:10.1002/mpr.1349

11.	Patalay P, Hardman CA. Comorbidity, Codevelopment, and Temporal Associations Between Body Mass Index and Internalizing Symptoms From Early Childhood to Adolescence. JAMA Psychiatry. 2019;76: 721–729. doi:10.1001/jamapsychiatry.2019.0169

12.	Sharpe H, Patalay P, Choo T-H, Wall M, Mason SM, Goldschmidt AB, et al. Bidirectional Associations between Body Dissatisfaction and Depressive Symptoms from Adolescence through Early Adulthood. Dev Psychopathol. 2018;30: 1447–1458. doi:10.1017/S0954579417001663

13.	Goodman R. Psychometric properties of the strengths and difficulties questionnaire. J Am Acad Child Adolesc Psychiatry. 2001;40: 1337–1345. doi:10.1097/00004583-200111000-00015

14.	Goodman A, Goodman R. Strengths and Difficulties Questionnaire as a Dimensional Measure of Child Mental Health. Journal of the American Academy of Child & Adolescent Psychiatry. 2009;48: 400–403. doi:10.1097/CHI.0b013e3181985068

15.	Scoring the 4-17 year old or 18+ versions of the SDQ. Available: file:///C:/Users/madel/Downloads/SDQ_English(UK)_4-17scoring%20(1).pdf

16.	Cole TJ, Bellizzi MC, Flegal KM, Dietz WH. Establishing a standard definition for child overweight and obesity worldwide: international survey. BMJ. 2000;320: 1240–1243. doi:10.1136/bmj.320.7244.1240

17.	World Health Organisation. Growth reference 5-19 years - BMI-for-age (5-19 years). 2023 [cited 15 Nov 2023]. Available: https://www.who.int/tools/growth-reference-data-for-5to19-years/indicators/bmi-for-age

18.	Ogden CL, Freedman DS, Hales CM. CDC Extended BMI-for-Age Percentiles Versus Percent of the 95th Percentile. Pediatrics. 2023;152: e2023062285. doi:10.1542/peds.2023-062285

19.	Heron JE, Croudace TJ, Barker ED, Tilling K. A comparison of approaches for assessing covariate effects in latent class analysis. Longitudinal and Life Course Studies. 2015;6: 420–434. doi:10.14301/llcs.v6i4.322
