# Supplementary material for: Associations of childhood body composition trajectories and adolescent body dissatisfaction: Longitudinal evidence from the UK Millennium Cohort Study
Source: PLoS One. 2026 Oct 14;21(10):e0339555. doi: 10.1371/journal.pone.0339555 (PMC13667782; doi:10.1371/journal.pone.0339555)
Supplement: Supplementary file 3 [file pone.0339555.s004.docx]

We thank the editor and reviewers for their careful consideration and constructive comments on our article *Associations of childhood body composition trajectories and adolescent body dissatisfaction: Longitudinal evidence from the UK Millennium Cohort Study*. We have revised the manuscript according to their feedback and provide details below of the changes made in response to each point raised. We have included in an updated submission a marked-up copy of the manuscript with tracked changes, as well as a clean, revised manuscript without tracked changes, and respond to reviewer comments below.

**Editor comments**

1. 1.Please ensure that your manuscript meets PLOS ONE's style requirements, including those for file naming.

**Response:** Thank you for signposting to the style templates. We have updated these throughout the manuscript in accordance with journal requirements.

2. Please expand the acronym “ESRC-BBSRC” (as indicated in your financial disclosure) so that it states the name of your funders in full. This information should be included in your cover letter; we will change the online submission form on your behalf.

**Response:** Thank you. For changes to the online submission, the joint funders are the Economic and Social Research Council (ESRC) and the Biotechnology and Biological Sciences Research Council (BBSRC).

3. Please include your full ethics statement in the ‘Methods’ section of your manuscript file. In your statement, please include the full name of the IRB or ethics committee who approved or waived your study, as well as whether or not you obtained informed written or verbal consent. If consent was waived for your study, please include this information in your statement as well.

**Response:** As we conducted a secondary data analysis of existing cohort data, a full ethics statement is not needed. However, we have clarified this is a secondary data analysis in the methods (page 5, line 101) and have included further detail about the ethical approval and consenting processes for the cohort (page 5, lines 105-108).

4. If the reviewer comments include a recommendation to cite specific previously published works, please review and evaluate these publications to determine whether they are relevant and should be cited. There is no requirement to cite these works unless the editor has indicated otherwise.

**Response:** We did not receive comments from reviewers to cite specific works.

**Reviewer 1**

1. Handling of implausible values and outliers

   The manuscript does not explicitly describe whether implausible anthropometric or body composition values were identified and excluded, or how extreme values were handled prior to trajectory modelling. As latent class growth analysis can be sensitive to outliers—particularly in defining high trajectory groups—it would be helpful for reproducibility to briefly clarify whether the analyses relied exclusively on standard Millennium Cohort Study data cleaning procedures or whether any additional checks were performed (e.g., biologically implausible value thresholds or distribution-based assessment of extreme observations).

**Response 1:** Thank you for this helpful suggestion. We have clarified how we handled potential outliers in the Exposure section (page 6, lines 135-136):
*“Range checks incorporated in interviewer scripts highlighted implausible values to improve accuracy of data collection. Additionally, we checked for extreme observations and excluded values which were more than 5 Standard Deviations above or below the study mean.”*

2. Interpretation of FFMI in relation to body dissatisfaction

The use of FFMI as a proxy for muscularity is appropriate and consistent with current epidemiological and clinical practice, particularly in large population-based studies where direct assessment of skeletal muscle mass is not feasible. There are no non-invasive methods that isolate muscle mass alone at scale, and FFMI remains the most widely accepted operational measure of fat-free mass in this context.

The observed associations between higher FFMI trajectories and greater body dissatisfaction—especially the absence of a protective association in boys—are therefore not a measurement issue, but an important interpretative finding. In childhood and adolescence, higher FFMI may reflect heterogeneous physiological profiles, including increased muscularity, greater bone mass, rapid linear growth during puberty, and adaptive increases in fat-free mass accompanying higher adiposity. These profiles may share similar FFMI values while carrying different social meanings and subjective body experiences.

It would strengthen the manuscript to make this interpretative distinction more explicit, emphasising that while FFMI is a valid proxy for fat-free mass, its psychosocial meaning may not be uniform across developmental stages or sexes. This would help avoid overly simplified readings of the findings and further highlight the contribution of the study.

**Response 2:** Thank you for this insightful comment. We have revised our discussion of FFMI results to incorporate this interpretation (page 17, lines 322-335):

*“High FFMI could capture a range of different physiological profiles, such as high muscularity (which we speculate could include those highly engaged across childhood in sports with a higher prevalence of eating disorders and body image concerns, or where leanness is considered a competitive advantage, e.g., gymnastics and swimming (38)), adaptive increases in lean mass as a result of higher adiposity (36), or rapid growth during puberty (39). It is plausible that high FFMI in these different scenarios may be associated with dissatisfaction with different aspects of body size or shape which are all captured by a global body dissatisfaction score in our study. Within this, there may be differences by sex in the aspects of body size and shape which contribute to overall body dissatisfaction, which we were not able to test (e.g., hypothetically, a female swimmer may have higher FFMI due to elevated lean mass, which they may not feel is socially desirable; a boy experiencing rapid increases in height during puberty may have high FFMI as a result of increased skeletal mass, but desire greater muscularity). Thus, our findings may suggest that high fat-free mass could have different social meanings and subjective body experiences which may vary across developmental stages and sexes.”*

3. Hypotheses and observed sex differences

The absence of sex differences in associations for adiposity trajectories, alongside the positive association between higher FFMI trajectories and body dissatisfaction in both sexes, represents an important and thought-provoking result. The Discussion could be further enriched by reflecting more explicitly on why theoretically grounded, gender-specific hypotheses may not fully apply in contemporary adolescent contexts. Consideration of evolving body ideals, changing sociocultural pressures, and the broader environment in which weight- and body-related messages circulate could add depth to the interpretation.

**Response 3:** Thank you for raising this interesting point. We have added discussion of the possible reasons for the absence of sex differences in adiposity trajectories (page 18, lines 351-356), as well as the observed sex differences in FFMI results (page 17, lines 329-333):

*“Other studies have found a greater magnitude of association among girls, but a positive association in both sexes (e.g., (24)). It is possible that earlier in adolescence there are differences by sex, but by mid-adolescence, as in our study, these differences may disappear, perhaps as more boys have entered puberty. The absence of sex differences in our study may suggest that both boys and girls are susceptible to body dissatisfaction, which may arise because of societal weight stigma although we did not test this mechanism in our study.”*

*“Within this, there may be differences by sex in the aspects of body size and shape which contribute to overall body dissatisfaction, which we were not able to test (e.g., hypothetically, a female swimmer may have higher FFMI due to elevated lean mass, which they may not feel is socially desirable; a boy experiencing rapid increases in height during puberty may have high FFMI as a result of increased skeletal mass, but desire greater muscularity).”*

4. Outcome measure and interpretation of body dissatisfaction

Body dissatisfaction is assessed using a single-item, global measure of satisfaction with appearance, which is widely used, validated, and appropriate for large cohort studies. This measure effectively captures overall appearance-related dissatisfaction.

At the same time, this item does not distinguish between specific dimensions of body image (e.g., dissatisfaction related to weight, body shape, size, or muscularity). This distinction is particularly relevant when interpreting sex-specific associations and findings related to FFMI. Adolescents may experience satisfaction with certain aspects of their bodies (such as strength or muscularity) alongside dissatisfaction with others, all of which are condensed into a single global response.

I would gently encourage the authors to revisit the wording of the Conclusions and/or further problematise this issue in the Discussion, explicitly acknowledging that the outcome captures global body dissatisfaction rather than dissatisfaction with specific body attributes. This clarification would add conceptual nuance and align the interpretation more closely with what the measure is able to capture.

**Response 4:** Thank you for raising these points. We have included discussion of the limitations of our body dissatisfaction measure (page 15, lines 281-288), as well as its implications for interpreting FFMI results (page 17, lines 327-333):

*“Body dissatisfaction was measured using a single item. Although this has not been validated against body dissatisfaction scales, it has been used widely in prior research (1,18,24,30). However, this item reflects a global satisfaction score and cannot distinguish dissatisfaction with specific aspects of body shape and size, which may be pertinent in understanding sex differences. For example, multidimensional body dissatisfaction scales, such as those used in other cohorts (31), may capture specific dissatisfaction with muscles. Adolescents may feel satisfied with some aspects of their appearance and not with others, though this nuance is not captured with our global score, limiting our interpretation.”*

*“It is plausible that high FFMI in these different scenarios may be associated with dissatisfaction with different aspects of body size or shape which are all captured by a global body dissatisfaction score in our study. Within this, there may be differences by sex in the aspects of body size and shape which contribute to overall body dissatisfaction, which we were not able to test (e.g., hypothetically, a female swimmer may have higher FFMI due to elevated lean mass, which they may not feel is socially desirable; a boy experiencing rapid increases in height during puberty may have high FFMI as a result of increased skeletal mass, but desire greater muscularity).”*

5. Reporting guidelines and transparency

The manuscript is clearly reported and aligns well with good reporting practice for observational cohort studies. While formal adherence to reporting guidelines is not a requirement of PLOS ONE, the use of structured frameworks such as STROBE has been increasingly encouraged within the scientific community to improve clarity, transparency, and reproducibility.

In this spirit, the authors may wish to explicitly reference STROBE recommendations for cohort studies, or consult the checklist to ensure that all relevant reporting elements are clearly addressed. In addition, given the use of routinely collected cohort data and the central role of latent trajectory modelling, consideration of complementary guidelines such as RECORD (for studies using routinely collected data) and GRoLTS (for latent trajectory analyses) could further strengthen the manuscript. These suggestions are offered as optional tools to enhance an already strong paper.

**Response 5:** Thank you for this helpful suggestion. We have made adjustments throughout the manuscript to ensure we are reporting in accordance with STROBE and GRoLTS checklists.

6. Tables and consistency check

I carefully reviewed the tables for internal consistency (including totals, percentages, and alignment with the text). Overall, the tables appear well constructed, internally consistent, and coherent with the analytical approach. Percentages sum appropriately within each category (with only minor rounding differences), and the distribution of participants across trajectory groups is consistent throughout. I noted one small inconsistency between the text and Table 3: for girls in the moderate FFMI trajectory, the text reports “0.36, 95% CI 0.36, 0.47”, whereas Table 3 reports a 95% CI of “0.26, 0.47”. This appears to be a typographical error in the text, as the point estimate and table values are otherwise consistent. Clarifying this would improve accuracy and consistency. Aside from this minor issue, I did not identify duplicated values, incorrect totals, or other inconsistencies across the tables.

**Response 6:** Thank you for pointing out the typographical error. We have corrected this on page 15, line 262.

**Reviewer 2**

- The abstract is vague and does not adequately summarize the objectives, methods, and main findings.

**Response 7:** We have revised the abstract to provide further detail which we believe improves clarity and better summarizes the objectives, methods and main findings. We included: rationale relating to gendered body ideals; further clarity on exposures, outcomes and methods; stating a lack of sex differences in adiposity associations as a key finding; discussion of adiposity-related associations and the need for further research using measures of muscularity.

- The introduction does not clearly establish the rationale for the study nor does it highlight its incremental contribution to the existing literature.

**Response 8:** We have amended the introduction to clarify the rationale/contribution to the literature (page 4, lines 87-91):

*“This knowledge may help in designing interventions to promote good mental health in adolescence, given the links between body dissatisfaction and subsequent mental health problems (2,3). Further, this may suggest limitations when using BMI as a proxy for adiposity, which is pertinent due to BMI’s ubiquity across research.”*

- The methods section lacks essential information, making it difficult to assess the internal validity and reproducibility of the analyses.

**Response 9:** We have made a number of revisions to the methods section in response to comments from other reviewers, which we hope will address these concerns (see responses 4 (handling of outliers in exposure measures), 12 (bioelectrical impedance description) and 13 (directed acyclic graph to outline confounder selection).

- Finally, the discussion is superficial and does not explore the theoretical and practical implications of the results enough.

**Response 10:** We have made substantial amendments to the discussion, particularly in light of specific comments from other reviewers, which we hope addresses this point (see responses 2 and 14 (range of physiological profiles captured by FFMI, interpretation in light of global body dissatisfaction measure and potential different social meanings by sex and developmental stage); 3 and 19 (interpretations and implications of lack of sex differences in adiposity associations); 21 (effect sizes and clinical relevance); and more expansive discussion of limitations per responses 4 and 15 (single global body dissatisfaction measure); 16 (potential misclassification of trajectory group); and 20 (missing data).

**Reviewer 3**

Abstract
• Please check the upper limit of the confidence interval for the association between the high FFMI trajectory and body dissatisfaction in boys (line 35), as it appears to contain a typographical error.

**Response 11:** Thank you for drawing our attention to the error; we have corrected this (page 2, line 38).

Methods
• The description of bioelectrical impedance analysis (BIA) would benefit from further clarification. Although the Supplement indicates that the Tanita BF-522W device uses manufacturer-derived equations based on large multiethnic studies, it is unclear whether these included children and adolescents and whether the embedded equations have been validated for a population comparable to this cohort. The specific BIA model used should be reported in the Methods section (not only in the Supplement), and it should be clearly stated that body composition was derived using the device-specific prediction equation. If population-specific validation of this equation is limited, this should be acknowledged in the Limitations section as a potential source of measurement error in the exposure.

**Response 12:** Thank you for these helpful comments. We have moved details regarding BIA from the supplement into the main manuscript (page 6, lines 122-137) and have included more expansive discussion of limited device-specific validation in the Discussion (page 16, lines 290-293):

*“Body composition data was measured using bioelectrical impedance analysis, rather than the gold standard dual x-ray absorptiometry scans. Our body composition estimates from bioelectrical impedance analysis are based on manufacturer-derived equations and device-specific validations appear to have largely been based on data from White children (e.g., (32)). However, studies have previously shown concordance between bioelectrical impedance analysis and dual x-ray absorptiometry scanning methods using diverse samples of adolescents (33,34).”*

• The selection of potential confounders is described as being based on prior evidence linking these variables to weight development and mental health outcomes. While this rationale is reasonable, the manuscript would benefit from a clearer articulation of the assumed causal framework underlying the adjustment strategy. The inclusion of a directed acyclic graph (DAG) in the supplementary material would enhance clarity regarding the rationale for the selected covariates.

**Response 13:** Thank you for this suggestion. We have included a DAG in the supplementary material to clarify our assumptions around the causal associations between exposure, outcome and confounders and to justify the chosen adjustment set.

Measurement and exposure considerations
• FFMI is used as a proxy for muscularity, but it is an indirect measure that likely conflates multiple phenotypes, including lean mass, bone mass, and adiposity-related increases in fat-free mass. This limitation complicates the interpretation of FFMI-related results, and the manuscript should explicitly acknowledge this when discussing associations with body dissatisfaction. Clarifying the empirical basis versus speculative explanations (e.g., sport participation or social ideals of muscularity) would strengthen the discussion. Additional descriptive information—such as mean BMI or FMI within FFMI classes by sex—could help illustrate this heterogeneity and improve interpretability.

**Response 14:** Thank you for these helpful comments. We have added further, more expansive discussion of the multiple phenotypes that could be captured by FFMI in the Discussion, as well as clarifying the speculative basis for these and other explanations of FFMI results (page 16, lines 295-301 and page 17, lines 319-340 – see below). We have additionally provided descriptives of mean BMI within BMI, FMI and FFMI trajectory classes by sex in the Supplement (STable 2, page 6).

*“As dual x-ray absorptiometry was not used, this limited our investigation of distinct components of body composition to fat and fat-free mass. We were unable to further break down these components, for instance into lean mass. In this study, fat-free mass is used as a proxy for lean mass, but factors other than lean mass may contribute to raised fat-free mass, such as elevated bone mass (35), or as an adaptation to elevated fat mass (36). As a result, higher FFMI trajectory groups may capture multiple phenotypes beside higher muscularity.”*

*“Based on theory of the gendered nature of sociocultural body ideals, we had expected to find that higher adiposity in girls and lower FFMI (which may indicate lower muscularity) in boys are associated with greater body dissatisfaction. Instead, we found that high FFMI was associated with greater body dissatisfaction in both sexes and that the magnitude of this association was greater in girls than in boys, consistent with a previous study (17). High FFMI could capture a range of different physiological profiles, such as high muscularity (which we speculate could include those highly engaged across childhood in sports with a higher prevalence of eating disorders and body image concerns, or where leanness is considered a competitive advantage, e.g., gymnastics and swimming (38)), adaptive increases in lean mass as a result of higher adiposity (36), or rapid growth during puberty (39). It is plausible that high FFMI in these different scenarios may be associated with dissatisfaction with different aspects of body size or shape which are all captured by a global body dissatisfaction score in our study. Within this, there may be differences by sex in the aspects of body size and shape which contribute to overall body dissatisfaction, which we were not able to test (e.g., hypothetically, a female swimmer may have higher FFMI due to elevated lean mass, which they may not feel is socially desirable; a boy experiencing rapid increases in height during puberty may have high FFMI as a result of increased skeletal mass, but desire greater muscularity). Thus, our findings may suggest that high fat-free mass could have different social meanings and subjective body experiences which may vary across developmental stages and sexes. This warrants further investigation using methods of measuring body composition which can distinguish lean mass from other contributors to high fat-free mass. Coupled with body dissatisfaction scales which evaluate different aspects of appearance, this may lead to greater understanding of different risk factors for body dissatisfaction in girls and boys.”*

• The use of a single-item measure to assess body dissatisfaction should be discussed in the Discussion section. Although such measures are widely used as indicators of body dissatisfaction in large cohort studies, they do not allow specification of the particular aspect underlying dissatisfaction (e.g., adiposity, muscularity, overall body size, or specific body regions). This lack of specificity may limit the interpretability of the findings.

**Response 15:** Thank you for raising this important limitation. We have included this in the limitations (page 15, lines 282-289), as well as incorporating into our interpretation of the findings (page 17, lines 328-334):

*“Body dissatisfaction was measured using a single item. Although this has not been validated against body dissatisfaction scales, it has been used widely in prior research (1,18,24,30). However, this item reflects a global satisfaction score and cannot distinguish dissatisfaction with specific aspects of body shape and size, which may be pertinent in understanding sex differences. For example, multidimensional body dissatisfaction scales, such as those used in other cohorts (31), may capture specific dissatisfaction with muscles. Adolescents may feel satisfied with some aspects of their appearance and not with others, though this nuance is not captured with our global score, limiting our interpretation.”*

*“It is plausible that high FFMI in these different scenarios may be associated with dissatisfaction with different aspects of body size or shape which are all captured by a global body dissatisfaction score in our study. Within this, there may be differences by sex in the aspects of body size and shape which contribute to overall body dissatisfaction, which we were not able to test (e.g., hypothetically, a female swimmer may have higher FFMI due to elevated lean mass, which they may not feel is socially desirable; a boy experiencing rapid increases in height during puberty may have high FFMI as a result of increased skeletal mass, but desire greater muscularity).”*

Analytical considerations
• Trajectory membership was assigned using modal class assignment, with entropy values around 0.7–0.8 indicating some classification uncertainty. This should be mentioned in the Discussion, as misclassification could slightly attenuate or bias associations with body dissatisfaction.

**Response 16:** Thank you for this suggestion. We have included this in the limitations (page 16, lines 302-305):

*“We assigned participants to their most likely trajectory group using modal class assignment. Although our entropy indices indicated adequate classification, there is likely to be some uncertainty and potential misclassification, particularly with FFMI trajectories where entropy values were lower, which may bias associations with body dissatisfaction.”*

Results and discussion
Table 1: Please check the last row “Managerial or professional” for BMI Trajectory. The values are currently 68.5 271 4.4.

**Response 17:** Thank you for noting this typographical error. We have corrected this in the table.

• Although the discussion of weight stigma and public health messaging provides useful context, these constructs were not directly measured in the study. The tone should therefore be adjusted to clarify that these interpretations are hypothetical rather than empirically tested mechanisms.

**Response 18:** Thank you for raising this point. We have included more cautious language to explicitly state that these constructs and mechanisms were not tested (page 18, lines 356-357 and page 19, lines 366-368):

*“The absence of sex differences in our study may suggest that both boys and girls are susceptible to body dissatisfaction, which may arise because of societal weight stigma although we did not test this mechanism in our study.”*

*“Although we did not measure this in our study, it is possible that these strategies’ focus on weight could contribute to increased stigma and lead to feelings of guilt and shame in children with higher weights.”*

• Given the substantial overlap between BMI and FMI trajectory membership, and the very similar magnitude of associations observed for both measures, it would be valuable for the authors to discuss whether, in this cohort, BMI trajectories may largely reflect adiposity patterns. Clarifying this point would strengthen the interpretation of the findings and help contextualize the relative contribution of BMI and FMI in relation to body dissatisfaction.

**Response 19:** Thank you for this helpful comment. We have included discussion of the potential meaning of the overlap between BMI and FMI trajectories and their similar associations with body dissatisfaction in the Discussion (page 18, lines 341-343:

*“We found considerable overlap between BMI and FMI trajectories, as well as in their associations with body dissatisfaction in adolescence. This may suggest that, within this cohort and at these ages, BMI trajectory largely approximates adiposity trajectory at the population level.”*

Missing data
• Missingness was socially patterned, with higher loss among socioeconomically disadvantaged and ethnic minority participants—groups that were also more likely to belong to higher adiposity trajectories. Although results from imputed and complete case analyses were similar, this differential attrition may have implications for the estimation of inequalities, which should be acknowledged as a potential limitation.

**Response 20:** Thank you for raising this point. We have included this in our limitations (page 17, lines 313-317):

*“Finally, although we imputed missing data, attrition was higher among disadvantaged groups, a greater proportion of whom had higher adiposity trajectories. We included auxiliary variables in our imputation model and found similar results using samples with imputed and complete data, however this patterning of missing data may have impacted our imputation and estimates of the association with body dissatisfaction.”*

Effect size and clinical relevance
• The manuscript does not discuss the magnitude of the observed associations. The mean differences in body dissatisfaction appear modest, and the authors should comment on their potential clinical or practical relevance.

**Response 21:** Thank you for suggesting this. We have included discussion of effect sizes and their implications at the population level (page 18, lines 343-347):

*“Effect sizes were fairly modest, representing approximately 12% higher body dissatisfaction scores in those with high BMI or FMI trajectories compared to those with low trajectories, given the 6-point scale used. However, these may translate to large shifts in the distribution of body dissatisfaction at the population level (40).”*
