# Supplementary material for: Associations of childhood body composition trajectories and adolescent body dissatisfaction: Longitudinal evidence from the UK Millennium Cohort Study
Source: PLoS One. 2026 Oct 14;21(10):e0339555. doi: 10.1371/journal.pone.0339555 (PMC13667782; doi:10.1371/journal.pone.0339555)
Supplement: Supplementary file 4 [file pone.0339555.s005.docx]

We thank the editor and reviewers for their careful consideration and constructive comments on our article *Associations of childhood body composition trajectories and adolescent body dissatisfaction: Longitudinal evidence from the UK Millennium Cohort Study*. We have revised the manuscript according to their feedback and provide details below of the changes made in response to each point raised. We have included in an updated submission a marked-up copy of the manuscript with tracked changes, as well as a clean, revised manuscript without tracked changes, and respond to reviewer comments below.

**Reviewer 1**

1. The discussion occasionally appears to interpret FFMI as a proxy for muscularity. Given that FFMI captures multiple components of fat-free mass beyond skeletal muscle, I encourage the authors to use slightly more cautious language and avoid equating FFMI directly with muscularity.

**Response:** Thank you for this helpful comment. We agree that FFMI is not a direct measure of muscularity and reflects multiple components of fat-free mass. We reviewed the discussion for instances where we interpreted FFMI as a proxy for muscularity and included more cautious language and explicitly advised against interpreting FFMI in this way (page 16, lines 298-302):

“In this study, we were interested in fat-free mass as it may partly reflect muscularity. However, factors other than lean mass may contribute to raised fat-free mass, such as elevated bone mass (32), or as an adaptation to elevated fat mass (33). As a result, higher FFMI trajectory groups are likely to capture multiple phenotypes beside higher muscularity and should therefore not be interpreted as reflecting the association of muscularity and body dissatisfaction.”

2. The outcome measure reflects global satisfaction with appearance rather than a multidimensional assessment of body dissatisfaction. Although this measure has been used in previous studies, I suggest acknowledging this distinction more explicitly in the limitations section.

**Response:** Thank you for the suggestion to expand this point. We have added explicit discussion that the outcome measure we have used is conceptually distinct from a multidimensional construct of body dissatisfaction (page 15, lines 281-290):

“Body dissatisfaction was measured using a single item measure of global appearance satisfaction. Although this has not been validated against body dissatisfaction scales, it has been used widely in prior research as a proxy for body dissatisfaction (1,18,24,29). Nevertheless, global appearance dissatisfaction is conceptually distinct from the multidimensional construct of body dissatisfaction and cannot distinguish dissatisfaction with specific aspects of body shape or size, which may be particularly pertinent in understanding sex differences. For example, multidimensional body dissatisfaction scales, such as those used in other cohorts (30), can capture specific dissatisfaction with body fat or muscularity. Adolescents may feel satisfied with some aspects of their appearance and not with others, though this nuance is not captured with our global appearance dissatisfaction score, limiting our interpretation.”

3. Pubertal development may influence both body composition trajectories and body dissatisfaction during adolescence. The absence of adjustment for pubertal timing or maturation status should be discussed more explicitly as a potential source of residual confounding.

**Response:** Thank you for this suggestion. We have added explicit discussion of the potential role of pubertal status in the associations investigated, that we consider it to be a mediator given the childhood trajectories of body composition we modelled, but that it may have influenced late childhood and early adolescent body composition measures that were incorporated into trajectories (page 17, lines 313-318):

“As we modelled trajectories of body composition from early childhood, we could not control for pubertal status, as we considered it likely to be on the causal pathway given evidence that higher childhood adiposity is associated with earlier onset of puberty (35). Nevertheless, as the pubertal transition results in changes to body composition, this may have influenced measures of body composition in late childhood and early adolescence which we incorporated into our trajectory modelling.”

4. The conclusion that BMI may capture adiposity “well” could be slightly tempered. The findings support similar associations between BMI trajectories and BIA-derived FMI trajectories with body dissatisfaction but do not directly evaluate adiposity using criterion methods such as DXA.

**Response:** Thank you for highlighting this nuance. We have revised this sentence in the abstract to focus on the consistency of associations with body dissatisfaction when FMI is estimated with BIA (page 2, lines 39-40), and in the conclusion to refer to FMI, rather than to adiposity more generally (page 19, lines 375-377):

“BMI and FMI trajectories are similarly associated with body dissatisfaction when FMI is estimated using bioelectrical impedance analysis.”

“In this cohort, BMI trajectory approximated FMI trajectory and its association with body dissatisfaction, but FFMI trajectories were more distinct and their associations with body dissatisfaction differed among boys.”

5. This is a minor and entirely optional suggestion. Given the use of latent class growth analysis, the authors may wish to consider whether additional reporting aligned with selected recommendations from the GRoLTS (Guidelines for Reporting on Latent Trajectory Studies) checklist could further enhance transparency regarding model selection and class assignment procedures. In particular, reporting of classification diagnostics and model selection criteria may be useful for readers seeking to evaluate trajectory model performance. However, I do not consider this essential for publication, as the current reporting is generally adequate.

**Response:** Thank you for this suggestion. We appreciate the recommendation; as this was noted as an optional enhancement and the current reporting is considered generally adequate, we have elected not to make further changes with respect to this.

**Reviewer 3**

Methods:

6. I would encourage the authors to reconsider the following statement: “Fat tissue contains lower concentrations of water, and is therefore more resistant to conducting the electrical current, which allows the proportion of fat to be estimated.” (page 6, lines 128-130). This description may oversimplify the physiological and analytical basis of BIA. In general, impedance measurements are incorporated into prediction equations to estimate total body water and/or fat-free mass, from which fat mass is typically derived. However, because the equations used by the Tanita BF-522W are proprietary and not publicly available, the exact calculation process cannot be verified. I therefore suggest removing this mechanistic explanation.

**Response:** Thank you for this suggestion. We have removed the mechanistic explanation and highlighted the proprietary nature of the prediction equations used on page 6, lines 128-130:

“In bioelectrical impedance analysis, a weak electrical current is sent around the body from foot to foot to estimate the proportion of fat. Age, height and sex were recorded on the scales to aid estimation, calculated using proprietary equations determined by the manufacturer and based on large, multi-ethnic population studies.”

7. I would also reconsider the statement: “Data from bioelectrical impedance analysis has been shown to be highly correlated with dual x-ray absorptiometry (26)...” (page 6, lines 131-133). The cited reference evaluated a different BIA device and prediction equation, and therefore its findings may not be directly applicable to the Tanita BF-522W used in this cohort. I therefore suggest either providing validation evidence specific to this device (or closely related manufacturer equations) or removing this statement.

**Response:** Thank you for highlighting this oversight. We have removed the statement from the manuscript.

8. If the authors agree, the equations used to calculate BMI, FMI and FFMI could be incorporated directly into the Methods section rather than presented in a separate table. This would improve conciseness and avoid the need for a dedicated table presenting widely established formulas.

**Response:** Thank you for this suggestion. We have incorporated the equations directly into the Methods on page 6, lines 131-134:

“Using measured height and weight, we calculated BMI as $weight\div({height}^{2})$ and using body fat percentage we calculated fat mass as $(body fat percentage\div100)\times weight$ and fat-free mass as $weight-fat mass$, allowing us to estimate FMI as $fat mass\div\left( {height}^{2} \right)$ and FFMI as $fatfree mass\div{(height}^{2})$.”

Discussion:

9. Page 17 (line 320 -341): The discussion of FFMI has improved substantially. However, the hypothetical examples of swimmers and pubertal growth appear speculative, as these scenarios were not evaluated in the present study. I suggest simplifying this section and focusing on the broader interpretation that higher FFMI may reflect different underlying physiological characteristics associated with body dissatisfaction.

**Response:** Thank you for this feedback. We have removed the speculative examples to simplify the section and focus on the different physiological characteristics that may be associated with body dissatisfaction, particularly given the global score we used (page 17, lines 329-337:

“High FFMI could capture a range of different physiological profiles, such as high muscularity, adaptive increases in lean mass as a result of higher adiposity (33), or rapid growth during puberty (36). It is plausible that high FFMI in these different scenarios may be associated with dissatisfaction with different aspects of body size or shape which are all captured by a global body dissatisfaction score in our study. Within this, there may also be sex differences in the aspects of body size and shape which contribute to overall body dissatisfaction, which we were not able to test as a consequence of using a global dissatisfaction score. Thus, our findings may suggest that high fat-free mass could have different social meanings and subjective body experiences which may vary across developmental stages and sexes.”

Limitations:

10. "Our body composition estimates from bioelectrical impedance analysis are based on manufacturer-derived equations and device-specific validations appear to have largely been based on data from White children (e.g., (32)). However, studies have previously shown concordance between bioelectrical impedance analysis and dual x-ray absorptiometry scanning methods using diverse samples of adolescents (33,34)" (page 16, lines 292-294) - The cited references (33,34) evaluated different Tanita devices and prediction equations than the BF-522W used in the present study. Therefore, I do not think these studies adequately address the limitation discussed here. I suggest retaining the limitation regarding the use of manufacturer-derived equations, as there remains uncertainty regarding the applicability of these equations to the study population, and removing the subsequent statement regarding concordance with DXA.

**Response:** Thank you for this suggestion. We have removed the statement regarding concordance with DXA scans.

11. “As dual x-ray absorptiometry was not used, this limited our investigation of distinct
    components of body composition to fat and fat-free mass” (page 16, lines 296-298): The discussion appropriately acknowledges that FFMI is not a direct measure of muscularity. However, DXA would not directly measure muscle mass either; rather, it would allow further partitioning of fat-free mass into lean soft tissue and bone components. I suggest revising this statement for greater precision.

**Response:** Thank you for this suggestion. We have revised this statement on page 16, lines 294-298 to clarify what further information would be gained from DXA scans:

“As dual x-ray absorptiometry was not used, this limited our investigation of distinct components of body composition to fat and fat-free mass. We were unable to further break down fat-free mass, for instance into lean soft tissue and bone components which would be possible with dual x-ray absorptiometry.”
