# Supplementary material for: Associations of childhood body composition trajectories and adolescent body dissatisfaction: Longitudinal evidence from the UK Millennium Cohort Study
Source: PLoS One. 2026 Oct 14;21(10):e0339555. doi: 10.1371/journal.pone.0339555 (PMC13667782; doi:10.1371/journal.pone.0339555)
Supplement: Peer review history

# Peer review: Decision Letter 0

**DOI:** 10.1371/journal.pone.0339555.r001

**Authors:** Vitor Paravidino

8 Mar 2026

Dear Dr. Davies Kellock,

Thank you for submitting your manuscript to PLOS ONE. After careful consideration, we feel that it has merit but does not fully meet PLOS ONE’s publication criteria as it currently stands. Therefore, we invite you to submit a revised version of the manuscript that addresses the points raised during the review process.

Please submit your revised manuscript by Apr 22 2026 11:59PM. If you will need more time than this to complete your revisions, please reply to this message or contact the journal office at plosone@plos.org. When you're ready to submit your revision, log on to https://www.editorialmanager.com/pone/ and select the 'Submissions Needing Revision' folder to locate your manuscript file.

- A letter that responds to each point raised by the academic editor and reviewer(s). You should upload this letter as a separate file labeled 'Response to Reviewers'.
- A marked-up copy of your manuscript that highlights changes made to the original version. You should upload this as a separate file labeled 'Revised Manuscript with Track Changes'.
- An unmarked version of your revised paper without tracked changes. You should upload this as a separate file labeled 'Manuscript'.

If you would like to make changes to your financial disclosure, please include your updated statement in your cover letter. Guidelines for resubmitting your figure files are available below the reviewer comments at the end of this letter.

If applicable, we recommend that you deposit your laboratory protocols in protocols.io to enhance the reproducibility of your results. Protocols.io assigns your protocol its own identifier (DOI) so that it can be cited independently in the future. For instructions see: https://journals.plos.org/plosone/s/submission-guidelines#loc-laboratory-protocols. Additionally, PLOS ONE offers an option for publishing peer-reviewed Lab Protocol articles, which describe protocols hosted on protocols.io. Read more information on sharing protocols at https://plos.org/protocols?utm_medium=editorial-email&utm_source=authorletters&utm_campaign=protocols.

We look forward to receiving your revised manuscript.

Kind regards,

Vitor Barreto Paravidino

Academic Editor

PLOS One

Journal Requirements:

When submitting your revision, we need you to address these additional requirements.

1.Please ensure that your manuscript meets PLOS ONE's style requirements, including those for file naming. The PLOS ONE style templates can be found at

https://journals.plos.org/plosone/s/file?id=wjVg/PLOSOne_formatting_sample_main_body.pdf and

https://journals.plos.org/plosone/s/file?id=ba62/PLOSOne_formatting_sample_title_authors_affiliations.pdf

2. Please expand the acronym “ESRC-BBSRC” (as indicated in your financial disclosure) so that it states the name of your funders in full.

This information should be included in your cover letter; we will change the online submission form on your behalf.

3. Please include your full ethics statement in the ‘Methods’ section of your manuscript file. In your statement, please include the full name of the IRB or ethics committee who approved or waived your study, as well as whether or not you obtained informed written or verbal consent. If consent was waived for your study, please include this information in your statement as well.

4. If the reviewer comments include a recommendation to cite specific previously published works, please review and evaluate these publications to determine whether they are relevant and should be cited. There is no requirement to cite these works unless the editor has indicated otherwise.

[Note: HTML markup is below. Please do not edit.]

Reviewers' comments:

Reviewer's Responses to Questions

**Comments to the Author**

1. Is the manuscript technically sound, and do the data support the conclusions?

Reviewer #1: Yes

Reviewer #2: No

Reviewer #3: Yes

**********

2. Has the statistical analysis been performed appropriately and rigorously?

Reviewer #1: Yes

Reviewer #2: Yes

Reviewer #3: Yes

**********

3. Have the authors made all data underlying the findings in their manuscript fully available?

The PLOS Data policy

Reviewer #1: Yes

Reviewer #2: Yes

Reviewer #3: Yes

**********

4. Is the manuscript presented in an intelligible fashion and written in standard English?

Reviewer #1: Yes

Reviewer #2: Yes

Reviewer #3: Yes

**********

Reviewer #1: This manuscript addresses an important and timely question by examining longitudinal associations between childhood body composition trajectories and body dissatisfaction in adolescence, using data from the UK Millennium Cohort Study. The study is thoughtfully designed and makes a valuable contribution by explicitly distinguishing trajectories of adiposity (BMI and FMI) from fat-free mass (FFMI), moving beyond reliance on BMI alone and engaging with a nuanced and relevant literature.

The analyses are clearly described and reflect careful and rigorous work. The longitudinal design, large sample size, repeated objective measurements, and systematic consideration of confounding factors provide a strong foundation for the research questions addressed. The manuscript is well structured, written in clear and accessible English, and the results are presented in a transparent and coherent manner.

The comments below are offered in a constructive spirit, with the aim of strengthening transparency, conceptual clarity, and interpretability, rather than raising concerns about the validity of the study.

1. Handling of implausible values and outliers

The manuscript does not explicitly describe whether implausible anthropometric or body composition values were identified and excluded, or how extreme values were handled prior to trajectory modelling. As latent class growth analysis can be sensitive to outliers—particularly in defining high trajectory groups—it would be helpful for reproducibility to briefly clarify whether the analyses relied exclusively on standard Millennium Cohort Study data cleaning procedures or whether any additional checks were performed (e.g., biologically implausible value thresholds or distribution-based assessment of extreme observations).

2. Interpretation of FFMI in relation to body dissatisfaction

The use of FFMI as a proxy for muscularity is appropriate and consistent with current epidemiological and clinical practice, particularly in large population-based studies where direct assessment of skeletal muscle mass is not feasible. There are no non-invasive methods that isolate muscle mass alone at scale, and FFMI remains the most widely accepted operational measure of fat-free mass in this context.

The observed associations between higher FFMI trajectories and greater body dissatisfaction—especially the absence of a protective association in boys—are therefore not a measurement issue, but an important interpretative finding. In childhood and adolescence, higher FFMI may reflect heterogeneous physiological profiles, including increased muscularity, greater bone mass, rapid linear growth during puberty, and adaptive increases in fat-free mass accompanying higher adiposity. These profiles may share similar FFMI values while carrying different social meanings and subjective body experiences.

It would strengthen the manuscript to make this interpretative distinction more explicit, emphasising that while FFMI is a valid proxy for fat-free mass, its psychosocial meaning may not be uniform across developmental stages or sexes. This would help avoid overly simplified readings of the findings and further highlight the contribution of the study.

3. Hypotheses and observed sex differences

The absence of sex differences in associations for adiposity trajectories, alongside the positive association between higher FFMI trajectories and body dissatisfaction in both sexes, represents an important and thought-provoking result. The Discussion could be further enriched by reflecting more explicitly on why theoretically grounded, gender-specific hypotheses may not fully apply in contemporary adolescent contexts. Consideration of evolving body ideals, changing sociocultural pressures, and the broader environment in which weight- and body-related messages circulate could add depth to the interpretation.

4. Outcome measure and interpretation of body dissatisfaction

Body dissatisfaction is assessed using a single-item, global measure of satisfaction with appearance, which is widely used, validated, and appropriate for large cohort studies. This measure effectively captures overall appearance-related dissatisfaction.

At the same time, this item does not distinguish between specific dimensions of body image (e.g., dissatisfaction related to weight, body shape, size, or muscularity). This distinction is particularly relevant when interpreting sex-specific associations and findings related to FFMI. Adolescents may experience satisfaction with certain aspects of their bodies (such as strength or muscularity) alongside dissatisfaction with others, all of which are condensed into a single global response.

I would gently encourage the authors to revisit the wording of the Conclusions and/or further problematise this issue in the Discussion, explicitly acknowledging that the outcome captures global body dissatisfaction rather than dissatisfaction with specific body attributes. This clarification would add conceptual nuance and align the interpretation more closely with what the measure is able to capture.

5. Reporting guidelines and transparency

The manuscript is clearly reported and aligns well with good reporting practice for observational cohort studies. While formal adherence to reporting guidelines is not a requirement of PLOS ONE, the use of structured frameworks such as STROBE has been increasingly encouraged within the scientific community to improve clarity, transparency, and reproducibility.

In this spirit, the authors may wish to explicitly reference STROBE recommendations for cohort studies, or consult the checklist to ensure that all relevant reporting elements are clearly addressed. In addition, given the use of routinely collected cohort data and the central role of latent trajectory modelling, consideration of complementary guidelines such as RECORD (for studies using routinely collected data) and GRoLTS (for latent trajectory analyses) could further strengthen the manuscript. These suggestions are offered as optional tools to enhance an already strong paper.

6. Tables and consistency check

I carefully reviewed the tables for internal consistency (including totals, percentages, and alignment with the text). Overall, the tables appear well constructed, internally consistent, and coherent with the analytical approach. Percentages sum appropriately within each category (with only minor rounding differences), and the distribution of participants across trajectory groups is consistent throughout. I noted one small inconsistency between the text and Table 3: for girls in the moderate FFMI trajectory, the text reports “0.36, 95% CI 0.36, 0.47”, whereas Table 3 reports a 95% CI of “0.26, 0.47”. This appears to be a typographical error in the text, as the point estimate and table values are otherwise consistent. Clarifying this would improve accuracy and consistency. Aside from this minor issue, I did not identify duplicated values, incorrect totals, or other inconsistencies across the tables.

Overall, this is a carefully conducted study, and I hope the comments above are received in the constructive spirit in which they are intended, with the aim of further strengthening the clarity and interpretability of the work.

Reviewer #2: The manuscript investigates the association between body composition trajectories in childhood and body dissatisfaction in adolescence, using data from the UK Millennium Cohort Study. The topic is relevant, the longitudinal design is adequate, and statistical rigor is observed in the analyses. However, important limitations compromise the scientific contribution of the study.

The abstract is vague and does not adequately summarize the objectives, methods, and main findings. The introduction does not clearly establish the rationale for the study nor does it highlight its incremental contribution to the existing literature. The methods section lacks essential information, making it difficult to assess the internal validity and reproducibility of the analyses. Finally, the discussion is superficial and does not explore the theoretical and practical implications of the results enough.

Thus, the manuscript would require substantial conceptual and methodological revisions to meet publication standards.

Reviewer #3: This longitudinal study investigates whether childhood trajectories of BMI, FMI and FFMI are associated with body dissatisfaction at age 14. Using data from a large population-based cohort and latent class growth analysis (LCGA), the authors report that higher BMI and FMI trajectories are associated with greater adolescent body dissatisfaction in both sexes, while FFMI shows sex-specific patterns.

Overall, the study addresses an important public health question, uses a large sample, and applies generally appropriate analytical methods. The manuscript is clearly written, and the supplementary material substantially improves transparency. However, several methodological aspects require clarification, and some points in the discussion and limitations would benefit from major revision—including exposure measurement, confounder selection, trajectory classification, interpretation of FFMI results, missing data, and effect sizes—as outlined below.

Abstract

• Please check the upper limit of the confidence interval for the association between the high FFMI trajectory and body dissatisfaction in boys (line 35), as it appears to contain a typographical error.

Methods

• The description of bioelectrical impedance analysis (BIA) would benefit from further clarification. Although the Supplement indicates that the Tanita BF-522W device uses manufacturer-derived equations based on large multiethnic studies, it is unclear whether these included children and adolescents and whether the embedded equations have been validated for a population comparable to this cohort. The specific BIA model used should be reported in the Methods section (not only in the Supplement), and it should be clearly stated that body composition was derived using the device-specific prediction equation. If population-specific validation of this equation is limited, this should be acknowledged in the Limitations section as a potential source of measurement error in the exposure.

• The selection of potential confounders is described as being based on prior evidence linking these variables to weight development and mental health outcomes. While this rationale is reasonable, the manuscript would benefit from a clearer articulation of the assumed causal framework underlying the adjustment strategy. The inclusion of a directed acyclic graph (DAG) in the supplementary material would enhance clarity regarding the rationale for the selected covariates.

Measurement and exposure considerations

• FFMI is used as a proxy for muscularity, but it is an indirect measure that likely conflates multiple phenotypes, including lean mass, bone mass, and adiposity-related increases in fat-free mass. This limitation complicates the interpretation of FFMI-related results, and the manuscript should explicitly acknowledge this when discussing associations with body dissatisfaction. Clarifying the empirical basis versus speculative explanations (e.g., sport participation or social ideals of muscularity) would strengthen the discussion. Additional descriptive information—such as mean BMI or FMI within FFMI classes by sex—could help illustrate this heterogeneity and improve interpretability.

• The use of a single-item measure to assess body dissatisfaction should be discussed in the Discussion section. Although such measures are widely used as indicators of body dissatisfaction in large cohort studies, they do not allow specification of the particular aspect underlying dissatisfaction (e.g., adiposity, muscularity, overall body size, or specific body regions). This lack of specificity may limit the interpretability of the findings.

Analytical considerations

• Trajectory membership was assigned using modal class assignment, with entropy values around 0.7–0.8 indicating some classification uncertainty. This should be mentioned in the Discussion, as misclassification could slightly attenuate or bias associations with body dissatisfaction.

Results and discussion

Table 1: Please check the last row “Managerial or professional” for BMI Trajectory. The values are currently 68.5 271 4.4.

• Although the discussion of weight stigma and public health messaging provides useful context, these constructs were not directly measured in the study. The tone should therefore be adjusted to clarify that these interpretations are hypothetical rather than empirically tested mechanisms.

• Given the substantial overlap between BMI and FMI trajectory membership, and the very similar magnitude of associations observed for both measures, it would be valuable for the authors to discuss whether, in this cohort, BMI trajectories may largely reflect adiposity patterns. Clarifying this point would strengthen the interpretation of the findings and help contextualize the relative contribution of BMI and FMI in relation to body dissatisfaction.

Missing data

• Missingness was socially patterned, with higher loss among socioeconomically disadvantaged and ethnic minority participants—groups that were also more likely to belong to higher adiposity trajectories. Although results from imputed and complete case analyses were similar, this differential attrition may have implications for the estimation of inequalities, which should be acknowledged as a potential limitation.

Effect size and clinical relevance

• The manuscript does not discuss the magnitude of the observed associations. The mean differences in body dissatisfaction appear modest, and the authors should comment on their potential clinical or practical relevance.

**********

what does this mean?). If published, this will include your full peer review and any attached files.

If you choose “no”, your identity will remain anonymous but your review may still be made public.

**Do you want your identity to be public for this peer review?**  For information about this choice, including consent withdrawal, please see our Privacy Policy

Reviewer #1: **Yes:** Camila Medeiros da Silva Mazzeti

Reviewer #2: No

Reviewer #3: No

**********

[NOTE: If reviewer comments were submitted as an attachment file, they will be attached to this email and accessible via the submission site. Please log into your account, locate the manuscript record, and check for the action link "View Attachments". If this link does not appear, there are no attachment files.]

To ensure your figures meet our technical requirements, please review our figure guidelines: https://journals.plos.org/plosone/s/figures

You may also use PLOS’s free figure tool, NAAS, to help you prepare publication quality figures: https://journals.plos.org/plosone/s/figures#loc-tools-for-figure-preparation.

NAAS will assess whether your figures meet our technical requirements by comparing each figure against our figure specifications.

Attachment Submitted filename: Review_Comments_to_the_Author_PLOS_ONE.pdf

# Author comment: Author response to Decision Letter 1

**DOI:** 10.1371/journal.pone.0339555.r002

1 Jun 2026

Please see uploaded document (Response to Reviewers)

Attachment Submitted filename: Response to Reviewers.docx

# Peer review: Decision Letter 1

**DOI:** 10.1371/journal.pone.0339555.r003

**Authors:** Vitor Paravidino

25 Jun 2026

Dear Dr. Davies Kellock,

Thank you for submitting your manuscript to PLOS ONE. After careful consideration, we feel that it has merit but does not fully meet PLOS ONE’s publication criteria as it currently stands. Therefore, we invite you to submit a revised version of the manuscript that addresses the points raised during the review process.

After evaluation by the reviewers and the editorial team, we believe that the manuscript has merit and is potentially suitable for publication. However, a few minor issues need to be addressed before a final decision can be made.

Please carefully consider the reviewers' comments and revise the manuscript accordingly. In your resubmission, provide a detailed response letter explaining how each comment was addressed, indicating the corresponding changes made in the manuscript.

Please submit your revised manuscript by Aug 09 2026 11:59PM. If you will need more time than this to complete your revisions, please reply to this message or contact the journal office at plosone@plos.org. When you're ready to submit your revision, log on to https://www.editorialmanager.com/pone/ and select the 'Submissions Needing Revision' folder to locate your manuscript file.

- A letter that responds to each point raised by the academic editor and reviewer(s). You should upload this letter as a separate file labeled 'Response to Reviewers'.
- A marked-up copy of your manuscript that highlights changes made to the original version. You should upload this as a separate file labeled 'Revised Manuscript with Track Changes'.
- An unmarked version of your revised paper without tracked changes. You should upload this as a separate file labeled 'Manuscript'.

If you would like to make changes to your financial disclosure, please include your updated statement in your cover letter. Guidelines for resubmitting your figure files are available below the reviewer comments at the end of this letter.

If applicable, we recommend that you deposit your laboratory protocols in protocols.io to enhance the reproducibility of your results. Protocols.io assigns your protocol its own identifier (DOI) so that it can be cited independently in the future. For instructions see: https://journals.plos.org/plosone/s/submission-guidelines#loc-laboratory-protocols. Additionally, PLOS ONE offers an option for publishing peer-reviewed Lab Protocol articles, which describe protocols hosted on protocols.io. Read more information on sharing protocols at https://plos.org/protocols?utm_medium=editorial-email&utm_source=authorletters&utm_campaign=protocols.

As the corresponding author, your ORCID iD is verified in the submission system and will appear in the published article. PLOS supports the use of ORCID, and we encourage all coauthors to register for an ORCID iD and use it as well. Please encourage your coauthors to verify their ORCID iD within the submission system before final acceptance, as unverified ORCID iDs will not appear in the published article. *Only*  the individual author can complete the verification step; PLOS staff *cannot*  verify ORCID iDs on behalf of authors.

We look forward to receiving your revised manuscript.

Kind regards,

Vitor Barreto Paravidino

Academic Editor

PLOS One

Journal Requirements:

If the reviewer comments include a recommendation to cite specific previously published works, please review and evaluate these publications to determine whether they are relevant and should be cited. There is no requirement to cite these works unless the editor has indicated otherwise.

Please review your reference list to ensure that it is complete and correct. If you have cited papers that have been retracted, please include the rationale for doing so in the manuscript text, or remove these references and replace them with relevant current references. Any changes to the reference list should be mentioned in the rebuttal letter that accompanies your revised manuscript. If you need to cite a retracted article, indicate the article’s retracted status in the References list and also include a citation and full reference for the retraction notice.

[Note: HTML markup is below. Please do not edit.]

Reviewers' comments:

Reviewer's Responses to Questions

**Comments to the Author**

Reviewer #1: All comments have been addressed

Reviewer #3: All comments have been addressed

**********

2. Is the manuscript technically sound, and do the data support the conclusions?

Reviewer #1: Yes

Reviewer #3: Yes

**********

3. Has the statistical analysis been performed appropriately and rigorously?

Reviewer #1: Yes

Reviewer #3: Yes

**********

4. Have the authors made all data underlying the findings in their manuscript fully available?

The PLOS Data policy

Reviewer #1: Yes

Reviewer #3: Yes

**********

5. Is the manuscript presented in an intelligible fashion and written in standard English?

Reviewer #1: Yes

Reviewer #3: Yes

**********

Reviewer #1: Thank you for the opportunity to review this manuscript. This is a well-conducted longitudinal study using a large, nationally representative cohort and objective measures of body composition. The research question is relevant, the analytical approach is appropriate, and the conclusions are generally supported by the data.

I have only a few minor comments that I believe would strengthen the manuscript:

The discussion occasionally appears to interpret FFMI as a proxy for muscularity. Given that FFMI captures multiple components of fat-free mass beyond skeletal muscle, I encourage the authors to use slightly more cautious language and avoid equating FFMI directly with muscularity.

The outcome measure reflects global satisfaction with appearance rather than a multidimensional assessment of body dissatisfaction. Although this measure has been used in previous studies, I suggest acknowledging this distinction more explicitly in the limitations section.

Pubertal development may influence both body composition trajectories and body dissatisfaction during adolescence. The absence of adjustment for pubertal timing or maturation status should be discussed more explicitly as a potential source of residual confounding.

The conclusion that BMI may capture adiposity “well” could be slightly tempered. The findings support similar associations between BMI trajectories and BIA-derived FMI trajectories with body dissatisfaction but do not directly evaluate adiposity using criterion methods such as DXA.

This is a minor and entirely optional suggestion. Given the use of latent class growth analysis, the authors may wish to consider whether additional reporting aligned with selected recommendations from the GRoLTS (Guidelines for Reporting on Latent Trajectory Studies) checklist could further enhance transparency regarding model selection and class assignment procedures. In particular, reporting of classification diagnostics and model selection criteria may be useful for readers seeking to evaluate trajectory model performance. However, I do not consider this essential for publication, as the current reporting is generally adequate.

Reference: van de Schoot R, Sijbrandij M, Winter SD, Depaoli S, Vermunt JK. The GRoLTS-Checklist: Guidelines for Reporting on Latent Trajectory Studies. Structural Equation Modeling. 2017;24(3):451–467. DOI: 10.1080/10705511.2016.1247646.

Overall, I believe this manuscript makes a valuable contribution to the literature and that these points primarily relate to clarification and interpretation rather than methodological concerns.

Reviewer #3: Thank you for addressing my previous comments. I believe the manuscript has improved substantially following revision. However, I have identified a few remaining points that I believe warrant further consideration, which I outline below.

Methods:

I would encourage the authors to reconsider the following statement: “Fat tissue contains lower concentrations of water, and is therefore more resistant to conducting the electrical current, which allows the proportion of fat to be estimated.” (page 6, lines 128-130). This description may oversimplify the physiological and analytical basis of BIA. In general, impedance measurements are incorporated into prediction equations to estimate total body water and/or fat-free mass, from which fat mass is typically derived. However, because the equations used by the Tanita BF-522W are proprietary and not publicly available, the exact calculation process cannot be verified. I therefore suggest removing this mechanistic explanation.

I would also reconsider the statement: “Data from bioelectrical impedance analysis has been shown to be highly correlated with dual x-ray absorptiometry (26)...” (page 6, lines 131-133). The cited reference evaluated a different BIA device and prediction equation, and therefore its findings may not be directly applicable to the Tanita BF-522W used in this cohort. I therefore suggest either providing validation evidence specific to this device (or closely related manufacturer equations) or removing this statement.

If the authors agree, the equations used to calculate BMI, FMI and FFMI could be incorporated directly into the Methods section rather than presented in a separate table. This would improve conciseness and avoid the need for a dedicated table presenting widely established formulas.

Discussion:

Page 17 (line 320 -341): The discussion of FFMI has improved substantially. However, the hypothetical examples of swimmers and pubertal growth appear speculative, as these scenarios were not evaluated in the present study. I suggest simplifying this section and focusing on the broader interpretation that higher FFMI may reflect different underlying physiological characteristics associated with body dissatisfaction.

Limitations:

"Our body composition estimates from bioelectrical impedance analysis are based on manufacturer-derived equations and device-specific validations appear to have largely been based on data from White children (e.g., (32)). However, studies have previously shown concordance between bioelectrical impedance analysis and dual x-ray absorptiometry scanning methods using diverse samples of adolescents (33,34)" (page 16, lines 292-294) - The cited references (33,34) evaluated different Tanita devices and prediction equations than the BF-522W used in the present study. Therefore, I do not think these studies adequately address the limitation discussed here. I suggest retaining the limitation regarding the use of manufacturer-derived equations, as there remains uncertainty regarding the applicability of these equations to the study population, and removing the subsequent statement regarding concordance with DXA.

“As dual x-ray absorptiometry was not used, this limited our investigation of distinct

components of body composition to fat and fat-free mass” (page 16, lines 296-298): The discussion appropriately acknowledges that FFMI is not a direct measure of muscularity. However, DXA would not directly measure muscle mass either; rather, it would allow further partitioning of fat-free mass into lean soft tissue and bone components. I suggest revising this statement for greater precision.

**********

what does this mean?). If published, this will include your full peer review and any attached files.

If you choose “no”, your identity will remain anonymous but your review may still be made public.

**Do you want your identity to be public for this peer review?**  For information about this choice, including consent withdrawal, please see our Privacy Policy

Reviewer #1: **Yes:** Camila Medeiros da Silva Mazzeti

Reviewer #3: No

**********

[NOTE: If reviewer comments were submitted as an attachment file, they will be attached to this email and accessible via the submission site. Please log into your account, locate the manuscript record, and check for the action link "View Attachments". If this link does not appear, there are no attachment files.]

To ensure your figures meet our technical requirements, please review our figure guidelines: https://journals.plos.org/plosone/s/figures

You may also use PLOS’s free figure tool, NAAS, to help you prepare publication quality figures: https://journals.plos.org/plosone/s/figures#loc-tools-for-figure-preparation.

NAAS will assess whether your figures meet our technical requirements by comparing each figure against our figure specifications.

# Author comment: Author response to Decision Letter 2

**DOI:** 10.1371/journal.pone.0339555.r004

4 Aug 2026

See document

Attachment Submitted filename: Response_to_Reviewers_auresp_2.docx

# Peer review: Decision Letter 2

**DOI:** 10.1371/journal.pone.0339555.r005

**Authors:** Vitor Paravidino

7 Sep 2026

Associations of childhood body composition trajectories and adolescent body dissatisfaction: Longitudinal evidence from the UK Millennium Cohort Study

PONE-D-25-65094R2

Dear Dr. Davies Kellock,

We’re pleased to inform you that your manuscript has been judged scientifically suitable for publication and will be formally accepted for publication once it meets all outstanding technical requirements.

Within one week, you’ll receive an e-mail detailing the required amendments. When these have been addressed, you’ll receive a formal acceptance letter and your manuscript will be scheduled for publication.

An invoice will be generated when your article is formally accepted. Please note, if your institution has a publishing partnership with PLOS and your article meets the relevant criteria, all or part of your publication costs will be covered. Please make sure your user information is up-to-date by logging into Editorial Manager at Editorial Manager® and clicking the ‘Update My Information' link at the top of the page. For questions related to billing, please contact billing support.

If your institution or institutions have a press office, please notify them about your upcoming paper to help maximize its impact. If they’ll be preparing press materials, please inform our press team as soon as possible -- no later than 48 hours after receiving the formal acceptance. Your manuscript will remain under strict press embargo until 2 pm Eastern Time on the date of publication. For more information, please contact onepress@plos.org.

Kind regards,

Vitor Barreto Paravidino

Academic Editor

PLOS One

Additional Editor Comments (optional):

Reviewers' comments:

Reviewer's Responses to Questions

**Comments to the Author**

Reviewer #1: All comments have been addressed

Reviewer #3: All comments have been addressed

**********

2. Is the manuscript technically sound, and do the data support the conclusions?

Reviewer #1: Yes

Reviewer #3: Yes

**********

3. Has the statistical analysis been performed appropriately and rigorously?

Reviewer #1: Yes

Reviewer #3: Yes

**********

4. Have the authors made all data underlying the findings in their manuscript fully available?

The PLOS Data policy

Reviewer #1: Yes

Reviewer #3: Yes

**********

5. Is the manuscript presented in an intelligible fashion and written in standard English?

Reviewer #1: Yes

Reviewer #3: Yes

**********

Reviewer #1: Thank you for the careful revision of the manuscript. The paper has improved substantially across the successive rounds of review, particularly in terms of methodological transparency, interpretation of FFMI, discussion of the limitations of the body dissatisfaction measure, and consideration of potential sources of bias. The manuscript is now clearly structured, methodologically sound, and written in appropriate and fluent English.

I consider the manuscript ready for publication. I only noted a few very minor editorial points that could be corrected during the final editing stage. First, in the Results section, the subheading refers once to “body satisfaction” rather than “body dissatisfaction”, which is the terminology used throughout the manuscript. Second, the statement that “most” participants had a parent educated to degree level or equivalent may be slightly imprecise, as the reported proportion is 42.2%; “the largest proportion” would be more accurate. Finally, the statement that the observed effect sizes represent “approximately 12% higher body dissatisfaction scores” could be phrased more precisely as approximately 12% of the total scale range.

These are minor wording issues only and do not affect the interpretation, validity, or overall quality of the study. In my view, the manuscript is suitable for publication in PLOS ONE.

Reviewer #3: (No Response)

**********

what does this mean?). If published, this will include your full peer review and any attached files.

If you choose “no”, your identity will remain anonymous but your review may still be made public.

**Do you want your identity to be public for this peer review?**  For information about this choice, including consent withdrawal, please see our Privacy Policy

Reviewer #1: **Yes:** Camila Medeiros da Silva Mazzeti

Reviewer #3: No

**********

# Editor report: Acceptance letter

**DOI:** 10.1371/journal.pone.0339555.r006

**Authors:** Vitor Paravidino

PONE-D-25-65094R2

PLOS One

Dear Dr. Davies Kellock,

I'm pleased to inform you that your manuscript has been deemed suitable for publication in PLOS One. Congratulations! Your manuscript is now being handed over to our production team.

At this stage, our production department will prepare your paper for publication. This includes ensuring the following:

* All references, tables, and figures are properly cited

* All relevant supporting information is included in the manuscript submission,

* There are no issues that prevent the paper from being properly typeset

You will receive further instructions from the production team, including instructions on how to review your proof when it is ready. Please keep in mind that we are working through a large volume of accepted articles, so please give us a few days to review your paper and let you know the next and final steps.

Lastly, if your institution or institutions have a press office, please let them know about your upcoming paper now to help maximize its impact. If they'll be preparing press materials, please inform our press team within the next 48 hours. Your manuscript will remain under strict press embargo until 2 pm Eastern Time on the date of publication. For more information, please contact onepress@plos.org.

You will receive an invoice from PLOS for your publication fee after your manuscript has reached the completed accept phase. If you receive an email requesting payment before acceptance or for any other service, this may be a phishing scheme. Learn how to identify phishing emails and protect your accounts at https://explore.plos.org/phishing.

If we can help with anything else, please email us at customercare@plos.org.

Thank you for submitting your work to PLOS One and supporting open access.

Kind regards,

PLOS One Editorial Office Staff

on behalf of

Dr. Vitor Barreto Paravidino

Academic Editor

PLOS One
